# The score after 10 years of registration of systematic review protocols

**DOI:** 10.1186/s13643-022-02053-9

**Published:** 2022-09-05

**Authors:** Kim van der Braak, Mona Ghannad, Claudia Orelio, Pauline Heus, Johanna A. A. Damen, René Spijker, Karen Robinson, Hans Lund, Lotty Hooft

**Affiliations:** 1 Cochrane Netherlands, Julius Center for Health Sciences and Primary Care, University Medical Centre Utrecht, Utrecht University, Utrecht, The Netherlands; 2 Research Support, Diakonessenhuis Utrecht, Bosboomstraat 1, 3582 KE Utrecht, The Netherlands; 3 Medical Library, Amsterdam Public Health, Amsterdam UMC, University of Amsterdam, Amsterdam, The Netherlands; 4 Division of General Internal Medicine, Department of Medicine, Johns Hopkins University School of Medicine, Baltimore, MD USA; 5 Section for Evidence-Based Practice, Department of Health and Functioning, Western Norway University of Applied Sciences, Bergen, Norway

**Keywords:** Protocol registration, Systematic review, Implementation, Open science

## Abstract

**Background:**

With the exponential growth of published systematic reviews (SR), there is a high potential for overlapping and redundant duplication of work. Prospective protocol registration gives the opportunity to assess the added value of a new study or review, thereby potentially reducing research waste and simultaneously increasing transparency and research quality. The PROSPERO database for SR protocol registration was launched 10 years ago. This study aims to assess the proportion SRs of intervention studies with a protocol registration (or publication) and explore associations of SR characteristics with protocol registration status.

**Methods:**

PubMed was searched for SRs of human intervention studies published in January 2020 and January 2021. After random-stratified sampling and eligibility screening, data extraction on publication and journal characteristics, and protocol registration status, was performed. Both descriptive and multivariable comparative statistical analyses were performed.

**Results:**

A total of 357 SRs (2020: *n* = 163; 2021: *n* = 194) were included from a random sample of 1267 publications. Of the published SRs, 38% had a protocol. SRs that reported using PRISMA as a reporting guideline had higher odds of having a protocol than publications that did not report PRISMA (*OR* 2.71; 95% *CI*: 1.21 to 6.09). SRs with a higher journal impact factor had higher odds of having a protocol (*OR* 1.12; 95% *CI* 1.04 to 1.25). Publications from Asia had a lower odds of having a protocol (*OR* 0.43; 95% *CI* 0.23 to 0.80, reference category = Europe). Of the 33 SRs published in journals that endorse PROSPERO, 45% did not have a protocol. Most SR protocols were registered in PROSPERO (*n* = 129; 96%).

**Conclusions:**

We found that 38% of recently published SRs of interventions reported a registered or published protocol. Protocol registration was significantly associated with a higher impact factor of the journal publishing the SR and a more frequent self-reported use of the PRISMA guidelines. In some parts of the world, SR protocols are more often registered or published than others. To guide strategies to increase the uptake of SR protocol registration, further research is needed to gain understanding of the benefits and informativeness of SRs protocols among different stakeholders.

**Systematic review registration:**

osf.io/9kj7r/

**Supplementary Information:**

The online version contains supplementary material available at 10.1186/s13643-022-02053-9.

## Introduction

Events following the coronavirus disease 2019 (COVID-19) pandemic have underlined the realization that societies form interconnected networks that are part of a global community. This is a realization that the academic community has embraced for several decades [1]. Collecting and reporting data in a transparent manner, as well as sharing data through collaborative networks, belong to the fundamental principles of open science [2]. Closely related is the open access movement, which promotes free availability of research output. Both open science and open access aim to improve research quality, research data integrity, research data usage efficiency, and societal impact and implementation of academic research [3, 4].

Prospective protocol registration in publicly accessible repositories increases transparency and research quality. A priori protocols help researchers to prepare the research process, to choose appropriate methods, and to specify the research questions and outcomes beforehand [5]. Registration of protocols facilitates thorough checking of review methods and reduces unnecessary duplication of research by independent researchers [6, 7]. Furthermore, risk of bias is minimized, and potential academic misconduct might be prevented [4]. Besides these benefits, prospective protocol registration also serves clinicians, patients, and policymakers. Clinical trial registration (e.g., ClinicalTrials.gov) informs patients and clinicians about novel clinical interventions in the investigation phase [8]. Also, trial registration may help to prevent cover up of non-favorable clinical outcomes, thereby reducing publication bias and increasing patient safety [4, 6].

In the era of evidence-based medicine, a number of organizations, including Cochrane, the Campbell Collaboration, and the Joanna Briggs Institute have served the research and clinical community with high-quality evidence syntheses. These organizations were among the first to publish protocols for systematic reviews (SRs). Although Cochrane reviews constitute a small fraction (7%) of all published SRs [9], reviews from this organization are considered the “gold standard” [10]. A recent study reported that on average 80 systematic reviews were published per day in 2019 [9]. Other research has found that 67% of meta-analyses are overlapping, within one of the topics, a total of 13 overlapping meta-analyses [11]. This underscores the necessity of protocol registration for non-Cochrane reviews, which could potentially reduce research overlap and duplication and thereby research waste and waste of public finance [7, 12]. For this, the Prospective Register of Systematic Reviews (PROSPERO) was launched in 2011, allowing researchers from various disciplines to register protocols for SRs related to health care [10, 13]. Since then, other registries such as the Open Science Framework have become available. Besides registration, SR protocols can be published in peer-reviewed journals. Main advantage of protocol registration in online registries is easy accessibility, while protocol publication could benefit from improvements in methodological quality suggested by reviewers during the protocol peer-review process.

Since the launch of PROSPERO, the number of protocol registrations for systematic reviews is increasing [10, 13–15]. Several studies have assessed SR protocol registration [10, 13–21]. Some studies assessed protocol records [10, 16] or assessed whether a protocol record resulted in a published SR [13]. Other studies assessed the status of SR protocol registration from the onset of PROSPERO in various research areas (e.g., dentistry, environmental science) or type of review (e.g., diagnostic, intervention, prognostic) [14, 15, 17–21]. Three studies showed an increase in the proportion of SR protocol registration in time [14, 15, 20]. Noteworthy is that in one study, which included both Cochrane and non-Cochrane reviews, most SRs with registered protocols were Cochrane reviews [17]. Here, we investigated the proportion of SRs of intervention studies that have a registered protocol 10 years since the launch of PROSPERO.

### Objectives

This study aims to assess the proportion SRs of intervention studies which have a registered or published protocol 10 years since the launch of PROSPERO. In addition, to gain more insight into factors associated with having a SR protocol, we explored the association of SR characteristics with protocol registration or publication status.

## Methods

The protocol of this study was preregistered on the Open Science Framework (OSF) on September 17, 2021: osf.io/9kj7r/ [22]. At the time of preregistration, we had carried out our literature search and piloted our selection process, but had not commenced data extraction and/or analysis. Reporting of this manuscript was informed by the Preferred Reporting Items for Systematic Reviews and Meta-Analysis (PRISMA) [23, 24].

### Information sources

PubMed was searched for systematic reviews of interventions using the search filter by health-evidence Canada [25]. Details on the search strategy are provided in Supplementary file 1, Table 1. The search was last carried out on July 2, 2021. From the records identified by the search strategy, two random samples of 10% were drawn using simple random-stratified sampling in EPPI-Reviewer [26].

**Table 1:** Characteristics of included articles

	All SRs	SR with protocol*	SR without protocol*	Cochrane reviews
**Total**, *n*/*N* (%)	357	135/357 (38)	216/357 (61)	6/357 (1)
**Year**, *n*/*N* (%) *2020*	163/357 (46)	54/163 (33)	106/163 (65)	3/163 (2)
*2021*	194/357 (54)	81/194 (42)	110/194 (57)	3/194 (1)
**COVID related – Yes**, N (%)	8 (2)	3 (2)	5 (2)	0 (0)
**No. of authors**, median (Q1–Q3)	5 (4–7)	5 (4–7)	5 (4–7)	5 (3–6)
**No. of affiliations**, median (Q1–Q3)	3 (2–5)	3 (2–5)	3 (2–5)	4 (3–6)
**Funding source,***N* (%)				
*No funding*	134 (38)	50 (37)	81 (38)	3 (50)
*Nonprofit*	116 (32)	45 (34)	68 (31)	3 (50)
*Not reported*	92 (26)	35 (26)	57 (27)	0 (0)
*Not clear*	7 (2)	3 (2)	5 (2)	0 (0)
*For profit*	7 (2)	2 (1)	5 (2)	0 (0)
**COI statement —** Yes, *N* (%)	342 (96)	126 (93)	210 (97)	6 (100)
**PRISMA reported^**, *N* (%)				
*Yes*	272 (76)	116 (87)	156 (72)	0 (0)
*No*	51 (14)	9 (7)	37 (17)	5 (83)
*Only in flow chart*	34 (10)	10 (7)	23 (11)	1 (17)
**Journal impact factor**, median (Q1–Q3)	3.4 (2.4–5.0)	3.8 (2.6–5.4)	3.2 (2.3–4.3)	9.3 (9.3–9.3)
**Journal endorses PROSPERO+**, *N* (%)	33 (9)	12 (9)	15 (7)	6 (100)

Abbreviations: *SR* systematic reviews, *COI* conflicts of interest, *PRISMA* Preferred Reporting Items for Systematic Reviews and Meta-Analysis, *SD* standard deviation. *Both protocol registration or protocol publication was counted as “SR with protocol.” More detailed information on protocol registration or publication is provided in “Results” section. ^“Yes” was scored when authors reported to have used PRISMA in the manuscript. “Only in flow chart” was scored, when PRISMA acronym was only mentioned in the name of the flow chart. If PRISMA was not reported and not mentioned in the flow chart, “no” was scored. +Journals identified from the website of PROSPERO [28], which includes all journals from the publishers BMC and BMJ. Journals that endorse PROSPERO are indicated in Supplementary file 2, Table 2

### Eligibility criteria

Systematic reviews of human intervention studies were eligible for inclusion in this study if “systematic review” or “meta-analysis” was stated in the title or abstract or if it was clear that it was a systematic review, such as a Cochrane review. The global COVID-19 pandemic increased time pressure on researchers to publish COVID-related research as quickly as possible. As a consequence, researchers could have omitted protocol registration in order to decrease SR processing time. To avoid selection bias of protocol registration due to the global COVID-19 pandemic, we included records published in January 2020 or January 2021. To allow for correction of bias, we have also extracted whether included SRs were COVID related. Either e-pub or journal publication date was used for selection, which did not match the indexed publication date in PubMed for some of the studies [27]. We had no restriction on language. Non-interventional reviews, literature reviews, updates of reviews, and umbrella reviews were excluded.

### Study selection and data collection

The selection process and data collection were performed consecutively by a single author (either KvdB, MG, or CO). First, title and abstract were screened. When potentially eligible, full text was obtained and further screened for inclusion. When all eligibility criteria were met, data extraction was performed by the same author. Difficulties in study selection or data extraction were discussed by the authors until agreement was reached. Study selection was randomly (approximately 10% of studies) checked by KvdB. Extracted data was checked by an independent second researcher (KvdB, CO, and DI).

Data were recorded in a piloted data extraction sheet in Microsoft Excel (version 16.54, 2021). We manually extracted data on the characteristics of the SR publications (publication year, number of authors, number of affiliations, funding, conflicts of interest statement, self-reported use of PRISMA guidelines, and COVID relatedness). Additionally, the following details regarding protocol registration or publication were extracted: database of protocol registration and wherein the article the protocol was mentioned (abstract, methods, or other location). We assessed whether an identifier or registration number of the protocol was reported and whether this number was correct, as this is crucial for protocol retrieval. The registration number was considered correct if it led to a corresponding protocol. We identified journals that endorsed PROSPERO (i.e., journals that specifically stated their support on the website of PROSPERO) [28] and assessed the number of SRs that were published in these journals. Individual journal author guidelines were not checked for PROSPERO endorsement.

From Web of Science and the Journal Citation Reports, data were automatically retrieved for the following: topic of the publication (Web of Science categories), journal, journal impact factor, and journal of published protocol (if applicable). As journals without indexation in the Science Citation Index Expanded (SCIE) or the Social Science Citation Index (SSCI) have no impact factor, it was considered to be equal to zero.

### Data synthesis and analysis

The primary outcome for this study was the proportion of SR publications with a protocol registration or publication. Characteristics of the reviews were quantified using descriptive statistics. Normality of the data was checked through visual inspection by two authors (KvdB, PH). We used frequencies and percentages for categorical variables. Mean and standard deviation were used for normally distributed continuous variables and median and interquartile range if data were skewed.

For our secondary objective, variables included in the multivariable logistic regression to explore the association with protocol registration or publication in non-Cochrane SRs were informed by previous research within our team [29, 30]. We assumed that protocol registration or publication was more likely in SRs with a higher number of authors and affiliations, a higher journal impact factor, a COI statement reported, and reported to using the PRISMA guidelines. We also assumed that there would be differences in protocol registration according to different types of funding, compared to no funding and different continents, for which we choose the Europe as a reference category. We included year of publication (reference category = 2020) in the multivariable analysis to control for potential differences between these years. All statistical analyses were conducted in RStudio [31], and figures were created using the ggplot2 package [32].

## Results

**Fig. 1:**
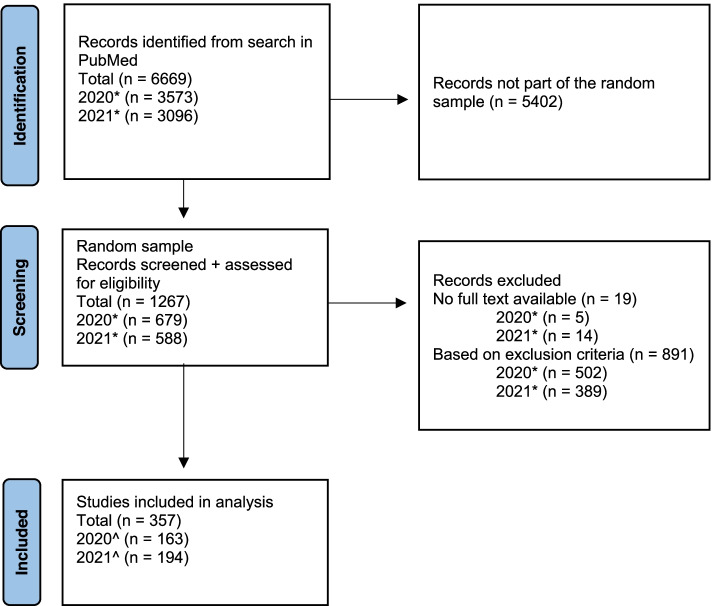
Preferred Reporting Items for Systematic Reviews and Meta-Analysis flow chart. *Indexed publication date in January 2020 or January 2021. ^Extracted publication date (either e-pub or journal publication date) in January 2020 or January 2021

A total of 6669 records were identified in our literature search (Fig. 1). A random sample of 1267 records was screened and assessed for eligibility. Reasons for exclusion were publication date (i.e., in months other than January), non-interventional studies (diagnostic or prognostic studies), or review types other than systematic review (e.g., updated review, scoping review) or nonhuman studies.

**Fig. 2:**
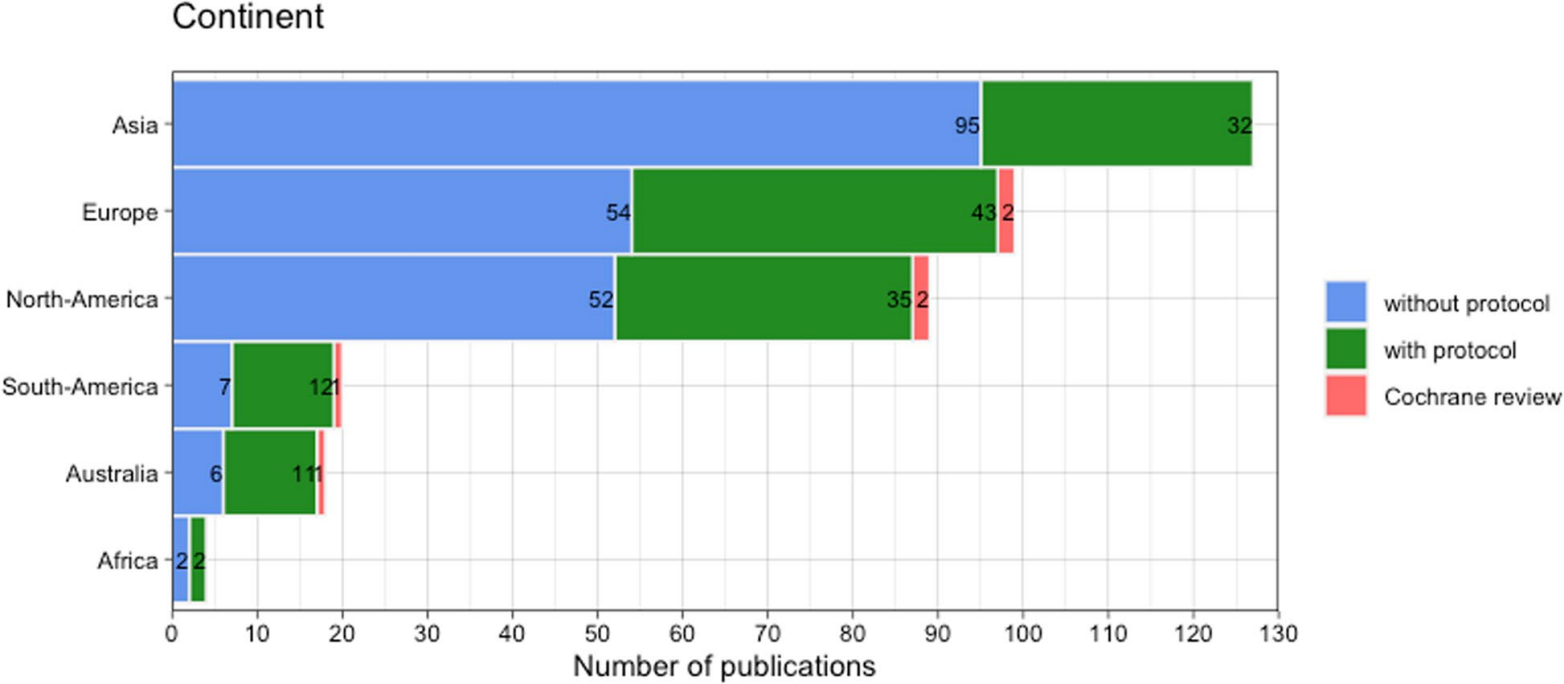
Continents of origin of the corresponding authors of all included SRs. The figure represents the total sample of SRs (*N* = 357)

**Fig. 3:**
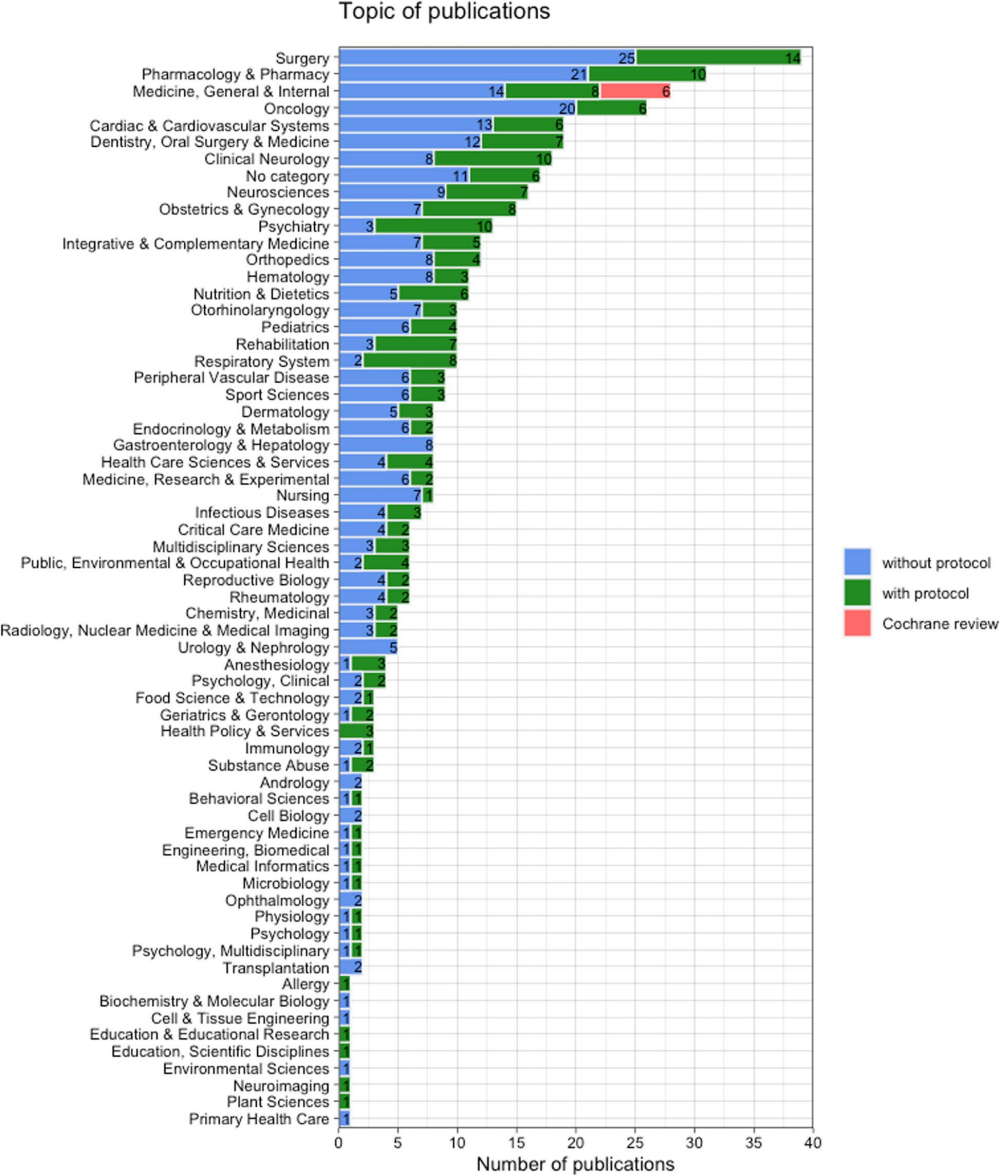
Topics of all included SRs. Topics are classified according to Web of Science (WoS) categories. No category was assigned to 17 included SRs that were not indexed to a WoS category. The figure represents the total sample of SRs (*N* = 357). As each SR could have been indexed to multiple topics, the total number exceeds the number of SRs

In total, 357 publications were included for analysis (*n* = 163 for January 2020 and *n* = 194 for January 2021). A reference list of included studies is provided in the Supplementary file 1. Characteristics of the included SRs are portrayed in Table 1. Overall, 96% of SRs reported a COI statement, 76% reported to use PRISMA guidelines, and most SRs were either not funded (*n* = 134, 38%) or had nonprofit funding (*n* = 116, 32%). A quarter of SRs did not report any information on funding (*n* = 92, 26%). The origin of the corresponding authors varied over 44 different countries and were mostly from Asia (*n* = 127, 36%), Europe (*n* = 99, 28%), and North America (*n* = 89, 25%) (Fig. 2 and Supplementary file 2, Fig. 1). The included SR publications also showed great variety in topics (*n* = 63) and journals in which the article was published (*n* = 280) (Fig. 3 and Supplementary file 2, Table 2). We identified 33 (9% of total sample) journals in our sample that explicitly endorses PROSPERO. For 17 publications, no topic category could be assigned. The three most frequent topics were surgery (*n* = 39), pharmacology and pharmacy and medicine (*n* = 31), and general and internal (*n* = 28).

**Table 2:** Characteristics of SRs with protocol registration

Items	With protocol registration (***N*** = 134) ***N*** (%)
**Where is protocol registration reported**	
*Abstract*	2 (1)
*Methods*	99 (74)*
*Abstract and methods*	29 (22)^
*Other*	4 (3)+
**Database of protocol registration**	
*PROSPERO*	129 (96)
*OSF*	3 (2)
*Other*	2 (2)
**Registration number reported, yes**	128 (96)

Abbreviations: *PROSPERO* Prospective Register of Systematic Reviews, *OSF* Open Science Framework. *Besides reporting protocol registration in the methods, one SR reported it in supplementary information as well, and 4 SRs reported registration on the title page as well. ^Besides reporting protocol registration in the abstract and methods, one SR reported it at the end of the article as well. +Protocol registration was only reported in the introduction (*n* = 1) or at the end of the article (*n* = 3). One SR, which was published and not registered, was excluded from Table 2 as the items reported in Table 2 are not applicable for publications

### Proportion of SRs with a protocol and differences with SRs without a protocol

Our sample included 6 (1%) Cochrane reviews (Table 1). Of the non-Cochrane reviews, 135 (38%) SRs had a protocol either registered, published, or both. The number of authors, affiliations, source of funding, and COVID-19 relatedness were similar between the SRs with and without a protocol. The percentage of SRs with a protocol was 42% in 2021 compared to 33% in 2020. Between the continents, percentages of SRs with a protocol varied from 25% (Asia) up to 61% (Australia). Several topic categories had ≥50% SR publications with a protocol, such as the following: clinical neurology, psychiatry, nutrition and dietetics, rehabilitation, respiratory system, and public, environmental, and occupational health. As there is a great variety in topics, only those with more than five publications are mentioned.

In SRs with a registered protocol, 86% reported to have applied the PRISMA guidelines. In contrast, in SR publications without protocol registration, the percentage that reported having used the PRISMA guidelines was 72%. The median journal impact factor was 3.8 (Q1–Q3, 2.6–5.4) for SRs with a protocol and 3.2 (Q1–Q3, 2.3–4.3) for those without a protocol. A total of 29 reviews were published in journals that were not indexed in the SCIE or SSCI. Of those reviews, 7 had a protocol, and 22 did not have a protocol. Of the SRs published in PROSPERO-endorsed journals, 45% (15 of 33) did not have a registered or published protocol.

The multivariable logistic regression (Supplementary file 2, Table 3) showed that the following factors were related to protocol registration or publication: self-reported use of PRISMA (reported vs. not reported), journal impact factor, and Asia as country of origin (versus Europe) (McFadden *R*^2^ = 0.096, *p* < 0.001). SR publications that reported using PRISMA guidelines have higher odds of having a protocol than publications that did not report using the PRISMA guidelines (*OR* 2.71; 95% *CI*: 1.21 to 6.09). SRs with a higher journal impact factor have a higher odds of having a protocol (*OR* 1.12; 95% *CI* 1.04 to 1.25). Publications from Asia had a lower odds of having a protocol (*OR* 0.43; 95% *CI* 0.23 to 0.80, reference category = Europe).

### Protocol registration or publication

In Cochrane reviews, protocol registration is reported in a specific section of the publication, in which deviations from the protocol are clarified. In line with Cochrane instructions, in the six Cochrane reviews from our study sample, protocol registration was not reported in the “Abstract” or “Methods” section of the publication, for which reason they are not presented in Table 2.

From the non-Cochrane review publications with a protocol (*n* = 135), 96% (*n* = 129) had protocol registration alone, and 4% (*n* = 5) had both registered and published their protocol. One SR had a published protocol and was excluded from Table 2, as it had no registration characteristics. Protocols were published in four different journals, and one protocol was posted on the National Institute for Health and Care Excellence (NICE) website (Supplementary file 2, Table 4).

Most SR publications reported protocol registration either in the “Methods” section (74%) or both in the abstract and methods (22%) (Table 2). Two SR publications reported information on protocol registration solely in the abstract (2%). Other sections in which protocol information was reported were as follows: at the end of the article (*n* = 4), on the title page (*n* = 4), the introduction (*n* = 1), or in the supplementary information (*n* = 1).

In our study, PROSPERO was predominantly used for protocol registration (*n* = 129; 96%). Other databases that were used are OSF (*n* = 3; 2%), the International Platform of Registered Systematic Review and Meta-Analysis Protocols (INPLASY) (*n* = 1; 1%), and the Research Registry (*n* = 1; 1%). As a correct identifier or registration number is key for protocol retrieval, we assessed the number of publications with a correct registration number. Almost all publications reported a registration number (*n* = 128; 96%), and most of those registration numbers were correct (*n* = 120; 94%). Incorrect registration numbers were either incomplete or the corresponding protocol could not be retrieved with the registration number reported. Of all registered SRs, 10% (*n* = 14) provided a direct URL to the protocol.

## Discussion

We found that 38% of recently published SRs of interventions reported a registered or published protocol, and 1% of our sample was Cochrane reviews. Protocol registration was significantly associated with a higher impact factor of the journal publishing the SR, a more frequent reporting of use of PRISMA guidelines and continent of origin.

Other studies have shown proportions of SRs with protocols ranging from 15 to 33% [14, 15, 17–21]. The proportion of 38% of SRs with a protocol found in our study aligns with the increasing trend over time [14, 15, 20]. Still, there seems to be a lot of variation in protocol registration proportion between studies. A study of environmental reviews observed 17% registered protocols in 2021 [18], while in the field of dentistry and orthodontics, 33–37% registered protocols were observed in 2017 [14, 19]. The differences in observed proportions of protocol registration between studies might not only be explained by the earlier publication date of study but also by the type of reviews (e.g., prognostic, diagnostic, intervention), the research topic, choice of examined registry, or a combination hereof.

Protocol registration seems to be positively linked with a higher methodological quality and improved reporting quality in systematic reviews [13, 17, 19]. In our study, protocol registration was significantly associated with a higher impact factor of the journal publishing the SR and a more frequent self-reported use of the PRISMA guidelines, which can be an indication of higher research quality. In journals with a higher impact factor (> 10), the risk of bias was found to be consistently lower with higher levels of registration of randomized controlled trials and the use of the CONSORT statement [30]. In contrast with Ge et al. (2018), we did not find an association between financial sponsorship and protocol registration. In line with Rombey et al. (2020), we observed that SRs from USA and China had low proportions, and that SRs from Canada and Australia had high proportions of SRs with registered protocols [15]. It is striking that, in our sample, Asia had the highest number of SRs published and the lowest proportion of registered protocols (25%).

In contrast to Allers et al. (2018), we observed that the number of SRs that reported the use of PRISMA was high for both SRs with a protocol (87%) and without a protocol (71%) [13]. A possible explanation is that the self-reported use of PRISMA reporting guidelines requires a smaller effort from review authors than protocol registration as reporting can be easily adjusted in hindsight, unlike protocol registration. Therefore, self-reported PRISMA use might have increased sharply in the last few years, also among authors that did not register a protocol. Hence, reporting guidelines can help to raise awareness for protocol registration among review authors, but reporting guidelines are insufficient to effectuate a priori protocol registration. This is supported by a recent survey study that showed that almost 45% of researchers, who have written a SR between 2010 and 2016, have never registered a SR protocol [33]. Most common reasons for not registering were as follows: lack of knowledge on benefits or process of protocol registration, lack of time, and nonmandatory requirement [33].

In 2005, the International Committee of Medical Journal Editors (ICMJE) has introduced mandatory trial registration guidelines for clinical trials on human subjects [34]. No such requirement exists for SRs. Even though some journals and organizations have endorsed protocol registration in PROSPERO, our results show that one-third of SRs published in these journals did not have a protocol. Hence, recommendations in author guidelines are insufficient to effectively implement prospective SR protocol registration. High-quality journals have the responsibility to uphold and continuously increase their quality standards to ensure high publication quality. Only after mandating registration of trials by the ICMJE, other journals followed, and the uptake of trial registration increased. Journal and publishers could fulfill a pioneer role in raising quality standards by making protocol registration a mandatory requirement for publication. As such, they could include assessment of protocol registration during the submission process or introduce a formal check as part of the peer-review process. A select number of journals already require protocol registration for SRs [35, 36]. Protocol registration awareness could be further enhanced by increasing or mandating adherence to the PRISMA for abstract reporting guidelines (reporting protocol registry name and number in the abstract) [37].

Some journals have adopted registered reports, which is similar to the Cochrane registration process, but is not exclusively aimed at SRs. During this process, research articles undergo peer review at the study design or protocol stage [38]. For Cochrane reviews, the scope of the proposed review and skills and experience of the proposed author team have typically been evaluated beforehand [39]. This provides an opportunity for feedback and support in the designing phase of the review to increase SR quality.

In the context of the open science movement, the scientific community and journals need to navigate between recommendation versus obligation of protocol registration to strike a balance between transparency and quality on the one hand and researchers’ time investment on the other. From the perspective of patients and guideline developers, high-quality, non-biased SRs are required for optimal clinical care and patient treatment for the reason that SRs and meta-analyses, more often than single clinical trials, directly inform medical guidelines and thereby medical treatment decisions.

Together, our results stress the need for further efforts to communicate the value of SR protocol registration more effectively, not only among researchers but also among journal editors and peer reviewers. To guide further efforts to highlight the value of SR protocol registration, research is needed to gain understanding of the benefits and informativeness of SRs protocol registration among different stakeholders (researchers, journal editors, peer reviewers, policy makers, the public, clinicians, patients).

### Strengths and limitations

A strength of our study is that we have assessed protocol registration from a sample of recently published SRs. However, a potential limitation is that some publications might not have reported protocol registration in their manuscript. Allers et al. found that 12.5% of SRs with a protocol did not report protocol registration or publication. We have not assessed whether this is the case in our sample and whether the proportion of interventional SRs with protocol registration we observed is an underestimation. When SR publications do not refer to the protocol, some of the benefits of protocol registration may be lost.

Another strength of our study is that it is unlikely that our results were influenced by COVID-related reviews. Not only have we selected our search periods to reflect both non-COVID and COVID pandemic times, but eventually, our sample had only a small proportion (2%) of COVID-related SRs.

Lastly, the generalizability of our study is limited, as we only included SRs of healthcare interventions. Protocol registration status might be different for other types of reviews (e.g., diagnostic, prognostic) and remains to be assessed in future research.

## Conclusions

We found that 135 out of 357 (38%) recently published interventional SRs had a protocol either registered or published. Our results did show that a higher journal impact factor and more frequent self-reported use of PRISMA guidelines was positively associated with protocol registration or publication. Noteworthy is that the continent with the highest number of published SRs (Asia) has the lowest proportion (25%) of SRs with protocol registration.

## Supplementary Information

**Additional file 1: Supplementary Table 1.** Search string

**Additional file 2: Supplementary Figure 1.** Countries of origen of the corresponding author. **Supplementary Table 2.** Frequency of included publications per journal title. **Supplementary Table 3.** outcomes of logistic regression. **Supplementary Table 4.** Frequency of SR protocols published in journals/ databases other than protocol registries.

## Data Availability

The data supporting the conclusions of this article is available in the Open Science Framework (OSF) repository: 10.17605/OSF.IO/TSUF9.

## Abbreviations

COI: Conflict of interest
INPLASY: International Platform of Registered Systematic Review and Meta-Analysis Protocols
JIF: Journal impact factor
NICE: National Institute for Health and Care Excellence
OSF: Open Science Framework
PRISMA: Preferred Reporting Items for Systematic Reviews and Meta-Analysis
PROSPERO: Prospective Register of Systematic Reviews
SCIE: Science Citation Index Expanded
SR: Systematic review
SSCI: Social Science Citation Index
