# Supplementary material for: The score after 10 years of registration of systematic review protocols
Source: Syst Rev. 2022 Sep 5;11:191. doi: 10.1186/s13643-022-02053-9 (PMC9444273; doi:10.1186/s13643-022-02053-9)
Supplement: Supplementary file 1 — Additional file 1: Supplementary Table 1. Search string [file 13643_2022_2053_MOESM1_ESM.docx]

Supplementary file 1

Supplementary table 1. Search string

((((systematic review[ti] OR systematic literature review[ti] OR systematic scoping review[ti] OR systematic narrative review[ti] OR systematic qualitative review[ti] OR systematic evidence review[ti] OR systematic quantitative review[ti] OR systematic meta-review[ti] OR systematic critical review[ti] OR

systematic mixed studies review[ti] OR systematic mapping review[ti] OR systematic cochrane review[ti] OR systematic search and review[ti] OR systematic integrative review[ti]) NOT comment[pt] NOT (protocol[ti] OR protocols[ti])) NOT MEDLINE [subset]) OR (Cochrane Database Syst Rev[ta] AND review[pt]) OR

systematic review[pt])

AND

((cohort[all] OR (control[all] AND study[all]) OR (control[tw] AND group*[tw]) OR epidemiologic studies[mh] OR program[tw] OR

clinical trial[pt] OR comparative stud*[all] OR evaluation studies[all] OR statistics as topic[mh] OR survey*[tw] OR follow-up*[all] OR

time factors[all] OR ci[tw]) NOT ((animals[mh:noexp] NOT humans[mh:noexp]) OR comment[pt] OR editorial[pt] OR review[pt] OR

meta analysis[pt] OR case report[tw] OR consensus[mh] OR guideline[pt] OR history[sh])) OR ((randomized controlled trial [pt] OR controlled clinical trial [pt] OR randomized [tiab] OR placebo [tiab] OR drug therapy [sh] OR randomly [tiab] OR trial [tiab] OR groups [tiab]) NOT (animals [mh] NOT humans [mh]))

Filters: from 2020/1/1 – 2020/1/31

Filters: from 2021/1/1 – 2021/1/31

References of included studies (n=357)

1. Abdelhaleem, N., et al., *Effect of action observation therapy on motor function in children with cerebral palsy: a systematic review of randomized controlled trials with meta-analysis.* CLINICAL REHABILITATION, 2021. **35**(1): p. 51-63.

2. Abou, L., et al., *Effectiveness of Physical Therapy Interventions in Reducing Fear of Falling Among Individuals With Neurologic Diseases: A Systematic Review and Meta-analysis.* ARCHIVES OF PHYSICAL MEDICINE AND REHABILITATION, 2021. **102**(1): p. 132-154.

3. Adam, F.A., et al., *Salvadora persica L. chewing stick and standard toothbrush as anti-plaque and anti-gingivitis tool: A systematic review and meta-analysis.* JOURNAL OF ETHNOPHARMACOLOGY, 2021. **274**.

4. Agasthi, P., et al., *Safety and efficacy of direct oral anticoagulants compared to Vitamin K antagonists postpercutaneous coronary interventions in patients with atrial fibrillation: A systematic review and meta-analysis.* JOURNAL OF ARRHYTHMIA, 2020. **36**(2): p. 271-279.

5. Ahmed, A.F., et al., *The safety of outpatient total shoulder arthroplasty: a systematic review and meta-analysis.* INTERNATIONAL ORTHOPAEDICS, 2021. **45**(3): p. 697-710.

6. Ahmed, S., et al., *Behaviour change techniques in personalised care planning for older people: a systematic review.* BRITISH JOURNAL OF GENERAL PRACTICE, 2021. **71**(703): p. E121-E127.

7. Akhlaghi, M., et al., *Effect of nuts on energy intake, hunger, and fullness, a systematic review and meta-analysis of randomized clinical trials.* CRITICAL REVIEWS IN FOOD SCIENCE AND NUTRITION, 2020. **60**(1): p. 84-93.

8. Al Sahlawi, M., et al., *Peritoneal dialysis-associated peritonitis outcomes reported in trials and observational studies: A systematic review.* PERITONEAL DIALYSIS INTERNATIONAL, 2020. **40**(2): p. 132-140.

9. Al-Abdouh, A., et al., *Complete Revascularization in Patients With STEMI and Multi-Vessel Disease: A Meta-Analysis of Randomized Controlled Trials.* CARDIOVASCULAR REVASCULARIZATION MEDICINE, 2020. **21**(5): p. 684-691.

10. Albrecht, E., et al., *Low- versus high-dose intraoperative opioids: A systematic review with meta-analyses and trial sequential analyses.* ACTA ANAESTHESIOLOGICA SCANDINAVICA, 2020. **64**(1): p. 6-22.

11. Aldaghlawi, F., et al., *A Systematic Review of Digital vs Analog Drainage for Air Leak After Surgical Resection or Spontaneous Pneumothorax.* CHEST, 2020. **157**(5): p. 1346-1353.

12. Amer, M.R., et al., *Outcomes of Transcarotid Versus Trans-Subclavian Transcatheter Aortic Valve Replacement: A Systematic Review and Meta-Analysis.* Cardiovasc Revasc Med, 2021. **33**: p. 20-25.

13. Amiri, N., M. Fathei, and M.M. Ziaaldini, *Effects of resistance training on muscle strength, insulin-like growth factor-1, and insulin-like growth factor-binding protein-3 in healthy elderly subjects: a systematic review and meta-analysis of randomized controlled trials.* HORMONES-INTERNATIONAL JOURNAL OF ENDOCRINOLOGY AND METABOLISM, 2021. **20**(2): p. 247-257.

14. Andani, A., et al., *One or two doses of hepatitis A vaccine in universal vaccination programs in children in 2020: A systematic review.* Vaccine, 2022. **40**(2): p. 196-205.

15. Asbaghi, O., et al., *Effect of green tea on glycemic control in patients with type 2 diabetes mellitus: A systematic review and meta-analysis.* DIABETES & METABOLIC SYNDROME-CLINICAL RESEARCH & REVIEWS, 2021. **15**(1): p. 23-31.

16. Askari, M., et al., *Effects of garlic supplementation on oxidative stress and antioxidative capacity biomarkers: A systematic review and meta-analysis of randomized controlled trials.* PHYTOTHERAPY RESEARCH, 2021. **35**(6): p. 3032-3045.

17. Avgerinos, I., et al., *Comparative efficacy and safety of glucose-lowering drugs as adjunctive therapy for adults with type 1 diabetes: A systematic review and network meta-analysis.* DIABETES OBESITY & METABOLISM, 2021. **23**(3): p. 822-831.

18. Aziz, M., et al., *Efficacy of Hemospray in non-variceal upper gastrointestinal bleeding: a systematic review with meta-analysis.* ANNALS OF GASTROENTEROLOGY, 2020. **33**(2): p. 145-+.

19. Azizi, N., et al., *The Effects of Nigella sativa Supplementation on Liver Enzymes Levels: a Systematic Review and Meta-analysis of Randomized Controlled Trials.* Clin Nutr Res, 2021. **10**(1): p. 72-82.

20. Baeza, M., et al., *Effect of periodontal treatment in patients with periodontitis and diabetes: systematic review and meta-analysis.* JOURNAL OF APPLIED ORAL SCIENCE, 2020. **28**.

21. Bahji, A., D. Carlone, and J. Altomare, *Acceptability and efficacy of naltrexone for criminal justice-involved individuals with opioid use disorder: a systematic review and meta-analysis.* ADDICTION, 2020. **115**(8): p. 1413-1425.

22. Bai, S., et al., *Efficacy and safety of anti-inflammatory agents for the treatment of major depressive disorder: a systematic review and meta-analysis of randomised controlled trials.* JOURNAL OF NEUROLOGY NEUROSURGERY AND PSYCHIATRY, 2020. **91**(1): p. 21-32.

23. Baili, E., et al., *Technical modifications and outcomes after Associating Liver Partition and Portal Vein Ligation for Staged Hepatectomy (ALPPS) for primary liver malignancies: A systematic review.* Surg Oncol, 2020. **33**: p. 70-80.

24. Bakdach, W.M.M. and R. Hadad, *EffPctiveness of low-level laser therapy in accelerating the orthodontic tooth movement: A systematic review and meta-analysis.* DENTAL AND MEDICAL PROBLEMS, 2020. **57**(1): p. 73-94.

25. Baniqued, P.D.E., et al., *Brain-computer interface robotics for hand rehabilitation after stroke: a systematic review.* JOURNAL OF NEUROENGINEERING AND REHABILITATION, 2021. **18**(1).

26. Bansal, V., et al., *Mortality Benefit of Remdesivir in COVID-19: A Systematic Review and Meta-Analysis.* FRONTIERS IN MEDICINE, 2021. **7**.

27. Barnett, M. and L. Reid, *The effectiveness of methylphenidate in improving cognition after brain injury in adults: a systematic review.* BRAIN INJURY, 2020. **34**(1): p. 1-10.

28. Belavy, D.L., et al., *Pain sensitivity is reduced by exercise training: Evidence from a systematic review and meta-analysis.* NEUROSCIENCE AND BIOBEHAVIORAL REVIEWS, 2021. **120**: p. 100-108.

29. Belk, J.W., et al., *Comparing Hamstring Autograft With Hybrid Graft for Anterior Cruciate Ligament Reconstruction: A Systematic Review.* ARTHROSCOPY-THE JOURNAL OF ARTHROSCOPIC AND RELATED SURGERY, 2020. **36**(4): p. 1189-1201.

30. Berthelsen, D.B., et al., *Harms reported by patients in rheumatology drug trials: a systematic review of randomized trials in the cochrane library from an OMERACT working group.* SEMINARS IN ARTHRITIS AND RHEUMATISM, 2021. **51**(3): p. 607-617.

31. Bezerra, R.P., et al., *HARTMANN PROCEDURE OR RESECTION WITH PRIMARY ANASTOMOSIS FOR TREATMENT OF PERFORATED DIVERTICULITIS? SYSTEMATIC REVIEW AND META-ANALYSIS.* ABCD-ARQUIVOS BRASILEIROS DE CIRURGIA DIGESTIVA-BRAZILIAN ARCHIVES OF DIGESTIVE SURGERY, 2020. **33**(3).

32. Bogani, G., et al., *Adjuvant chemotherapy vs. observation in stage I clear cell ovarian carcinoma: A systematic review and meta-analysis.* GYNECOLOGIC ONCOLOGY, 2020. **157**(1): p. 293-298.

33. Boldovjakova, D., et al., *Sublingual immunotherapy vs placebo in the management of grass pollen-induced allergic rhinitis in adults: A systematic review and meta-analysis.* CLINICAL OTOLARYNGOLOGY, 2021. **46**(1): p. 52-59.

34. Bovbjerg, P., et al., *Effect of PTH treatment on bone healing in insufficiency fractures of the pelvis: a systematic review.* EFORT OPEN REVIEWS, 2021. **6**(1): p. 9-14.

35. Brauer, P., D. Royall, and A. Rodrigues, *Use of the Healthy Eating Index in Intervention Studies for Cardiometabolic Risk Conditions: A Systematic Review.* ADVANCES IN NUTRITION, 2021. **12**(4): p. 1317-1331.

36. Brekke, A.F., et al., *Non-surgical interventions for excessive anterior pelvic tilt in symptomatic and non-symptomatic adults: a systematic review.* EFORT OPEN REVIEWS, 2020. **5**(1): p. 37-45.

37. Bule, M., et al., *The antidiabetic and antilipidemic effects of Hibiscus sabdariffa: A systematic review and meta-analysis of randomized clinical trials.* FOOD RESEARCH INTERNATIONAL, 2020. **130**.

38. Buneviciene, I., et al., *Can mHealth interventions improve quality of life of cancer patients? A systematic review and meta-analysis.* CRITICAL REVIEWS IN ONCOLOGY HEMATOLOGY, 2021. **157**.

39. Butterworth, R.F., *Beneficial effects of L-ornithine L-aspartate for prevention of overt hepatic encephalopathy in patients with cirrhosis: a systematic review with meta-analysis.* METABOLIC BRAIN DISEASE, 2020. **35**(1): p. 75-81.

40. Bzeizi, K.I., et al., *Long-Term Outcomes of Everolimus Therapy in De Novo Liver Transplantation: A Systematic Review and Meta-Analysis of Randomized Controlled Trials.* TRANSPLANTATION PROCEEDINGS, 2021. **53**(1): p. 148-158.

41. Campo, M., et al., *The effectiveness of biofeedback for improving pain, disability and work ability in adults with neck pain: A systematic review and meta-analysis.* MUSCULOSKELETAL SCIENCE AND PRACTICE, 2021. **52**.

42. Cao, J., et al., *The efficacy and safety of lamotrigine for absence seizures in children and adolescents: A systematic review and meta-analysis.* JOURNAL OF CLINICAL NEUROSCIENCE, 2020. **71**: p. 199-204.

43. Cardoso, P.C., et al., *Pain level between clear aligners and fixed appliances: a systematic review.* PROGRESS IN ORTHODONTICS, 2020. **21**(1).

44. Carrier, F.M., et al., *Restrictive fluid management strategies and outcomes in liver transplantation: a systematic review.* CANADIAN JOURNAL OF ANESTHESIA-JOURNAL CANADIEN D ANESTHESIE, 2020. **67**(1): p. 109-127.

45. Casadaban, L.C., J.C. Mandell, and Y. Epelboym, *Genicular Artery Embolization for Osteoarthritis Related Knee Pain: A Systematic Review and Qualitative Analysis of Clinical Outcomes.* CARDIOVASCULAR AND INTERVENTIONAL RADIOLOGY, 2021. **44**(1): p. 1-9.

46. Cerecedo-Lopez, C.D., et al., *Insulin in the Management of Acute Ischemic Stroke: A Systematic Review and Meta-Analysis.* WORLD NEUROSURGERY, 2020. **136**: p. E514-E534.

47. Chair, S.Y., H.J. Zou, and X. Cao, *Effects of Exercise Therapy for Adults With Coronary Heart Disease A Systematic Review and Meta-analysis of Randomized Controlled Trials.* JOURNAL OF CARDIOVASCULAR NURSING, 2021. **36**(1): p. 56-77.

48. Chan, J.K.Y., et al., *The effectiveness of e-interventions on fall, neuromuscular functions and quality of life in community-dwelling older adults: A systematic review and meta-analysis.* INTERNATIONAL JOURNAL OF NURSING STUDIES, 2021. **113**.

49. Chen, F., et al., *Efficacy of second-line treatments for patients with advanced human epidermal growth factor receptor 2 positive breast cancer after trastuzumab-based treatment: a systematic review and bayesian network analysis.* JOURNAL OF CANCER, 2021. **12**(6): p. 1687-1697.

50. Chen, H., M.L. Liang, and J. Min, *Efficacy and Safety of Bevacizumab-Combined Chemotherapy for Advanced and Recurrent Endometrial Cancer: A Systematic Review and Meta-analysis.* BALKAN MEDICAL JOURNAL, 2021. **38**(1): p. 7-+.

51. Chen, K., et al., *The Anti-PD-1/PD-L1 Immunotherapy for Gastric Esophageal Cancer: A Systematic Review and Meta-Analysis and Literature Review.* CANCER CONTROL, 2021. **28**.

52. Chen, L.Q., et al., *The Efficacy and Mechanism of Chinese Herbal Medicines in Lowering Serum Uric Acid Levels: A Systematic Review.* FRONTIERS IN PHARMACOLOGY, 2021. **11**.

53. Chen, M., et al., *Use of tyrosine kinase inhibitors for paediatric Philadelphia chromosome-positive acute lymphoblastic leukaemia: a systematic review and meta-analysis.* BMJ OPEN, 2021. **11**(1).

54. Chen, T., Z.Q. Zhu, and J.L. Du, *Efficacy of Intercostal Nerve Block for Pain Control After Percutaneous Nephrolithotomy: A Systematic Review and Meta-Analysis.* FRONTIERS IN SURGERY, 2021. **8**.

55. Chen, Y.F., et al., *Baseline HbA1c Level Influences the Effect of Periodontal Therapy on Glycemic Control in People with Type 2 Diabetes and Periodontitis: A Systematic Review on Randomized Controlled Trails.* DIABETES THERAPY, 2021. **12**(5): p. 1249-1278.

56. Chen, Y.Y., D.R. Chen, and H.C. Lin, *Infiltration and sealing for managing non-cavitated proximal lesions: a systematic review and meta-analysis.* BMC ORAL HEALTH, 2021. **21**(1).

57. Cheng, P.F., et al., *Role of Arts Therapy in Patients with Breast and Gynecological Cancers: A Systematic Review and Meta-Analysis.* JOURNAL OF PALLIATIVE MEDICINE, 2021. **24**(3): p. 443-452.

58. Chu, K.H., et al., *Smartphone health apps for tobacco Cessation: A systematic review.* ADDICTIVE BEHAVIORS, 2021. **112**.

59. Cicolo, E.A., et al., *Effectiveness of the Manchester Triage System on time to treatment in the emergency department: a systematic review.* JBI Evid Synth, 2020. **18**(1): p. 56-73.

60. Cifuentes, L.I., et al., *Band ligation versus sham or no intervention for primary prophylaxis of oesophageal variceal bleeding in children and adolescents with chronic liver disease or portal vein thrombosis.* COCHRANE DATABASE OF SYSTEMATIC REVIEWS, 2021(1).

61. Ciria, R., et al., *A systematic review and meta-analysis comparing the short- and long-term outcomes for laparoscopic and open liver resections for liver metastases from colorectal cancer.* SURGICAL ENDOSCOPY AND OTHER INTERVENTIONAL TECHNIQUES, 2020. **34**(1): p. 349-360.

62. Clement, W.A., et al., *Acute isolated sphenoid sinusitis in children: A case series and systematic review of the literature.* INTERNATIONAL JOURNAL OF PEDIATRIC OTORHINOLARYNGOLOGY, 2021. **140**.

63. Clunie, G.M., et al., *Voice and Swallowing Outcomes Following Airway Reconstruction in Adults: A Systematic Review.* LARYNGOSCOPE, 2021. **131**(1): p. 146-157.

64. Comelis, M.A., et al., *Treatment effect of bone-anchored maxillary protraction in growing patients compared to controls: a systematic review with meta-analysis.* EUROPEAN JOURNAL OF ORTHODONTICS, 2021. **43**(1): p. 51-68.

65. Conley, C.E.W., et al., *A Comparison of Neuromuscular Electrical Stimulation Parameters for Postoperative Quadriceps Strength in Patients After Knee Surgery: A Systematic Review.* SPORTS HEALTH-A MULTIDISCIPLINARY APPROACH, 2021. **13**(2): p. 116-127.

66. Cruz-Cobo, C. and M.J. Santi-Cano, *Efficacy of Diabetes Education in Adults With Diabetes Mellitus Type 2 in Primary Care: A Systematic Review.* JOURNAL OF NURSING SCHOLARSHIP, 2020. **52**(2): p. 155-163.

67. Cui, L., et al., *Effectiveness of progesterone-primed ovarian stimulation in assisted reproductive technology: a systematic review and meta-analysis.* ARCHIVES OF GYNECOLOGY AND OBSTETRICS, 2021. **303**(3): p. 615-630.

68. da Silva, B.C.L., et al., *Envelope or triangular flap for surgical removal of third molars? A systematic review and meta-analysis.* INTERNATIONAL JOURNAL OF ORAL AND MAXILLOFACIAL SURGERY, 2020. **49**(8): p. 1073-1086.

69. Dafni, U., et al., *Efficacy of cancer vaccines in selected gynaecological breast and ovarian cancers: A 20-year systematic review and meta-analysis.* EUROPEAN JOURNAL OF CANCER, 2021. **142**: p. 63-82.

70. Dai, W.L., et al., *Intra-Articular Mesenchymal Stromal Cell Injections Are No Different From Placebo in the Treatment of Knee Osteoarthritis: A Systematic Review and Meta-analysis of Randomized Controlled Trials.* ARTHROSCOPY-THE JOURNAL OF ARTHROSCOPIC AND RELATED SURGERY, 2021. **37**(1): p. 340-358.

71. Dalton-Locke, C., et al., *The Effectiveness of Mental Health Rehabilitation Services: A Systematic Review and Narrative Synthesis.* FRONTIERS IN PSYCHIATRY, 2021. **11**.

72. Davari, M., et al., *Pregabalin and gabapentin in neuropathic pain management after spinal cord injury: a systematic review and meta-analysis.* KOREAN JOURNAL OF PAIN, 2020. **33**(1): p. 3-12.

73. Davies, S.C., et al., *Oral Janus kinase inhibitors for maintenance of remission in ulcerative colitis.* COCHRANE DATABASE OF SYSTEMATIC REVIEWS, 2020(1).

74. Davies, T.B., et al., *Chronic Effects of Altering Resistance Training Set Configurations Using Cluster Sets: A Systematic Review and Meta-Analysis.* SPORTS MEDICINE, 2021. **51**(4): p. 707-736.

75. de Almeida, R.B.S., et al., *Surgical Treatment Applied to Bilateral Vocal Fold Paralysis in Adults: Systematic Review.* J Voice, 2021.

76. Delpino, F.M. and L.M. Figueiredo, *Resveratrol supplementation and type 2 diabetes: a systematic review and meta-analysis.* CRITICAL REVIEWS IN FOOD SCIENCE AND NUTRITION.

77. Diep, D., K.J.Q. Chen, and D. Kumbhare, *Ultrasound-guided interventional procedures for myofascial trigger points: a systematic review.* REGIONAL ANESTHESIA AND PAIN MEDICINE, 2021. **46**(1): p. 73-80.

78. Ding, F., et al., *The effects of green coffee bean extract supplementation on lipid profile in humans: A systematic review and meta-analysis of randomized controlled trials.* NUTRITION METABOLISM AND CARDIOVASCULAR DISEASES, 2020. **30**(1): p. 1-10.

79. Ding, Y.Q., Z.F. Chen, and Y.L. Lu, *Vitamin A supplementation prevents the bronchopulmonary dysplasia in premature infants A systematic review and meta-analysis.* MEDICINE, 2021. **100**(3).

80. Dludla, P.V., et al., *Adipokines as a therapeutic target by metformin to improve metabolic function: A systematic review of randomized controlled trials.* PHARMACOLOGICAL RESEARCH, 2021. **163**.

81. Duan, H.Z., et al., *Statin use and risk of tuberculosis: a systemic review of observational studies.* INTERNATIONAL JOURNAL OF INFECTIOUS DISEASES, 2020. **93**: p. 168-174.

82. Edmunds, K., et al., *Incidence of the adverse effects of androgen deprivation therapy for prostate cancer: a systematic literature review.* SUPPORTIVE CARE IN CANCER, 2020. **28**(5): p. 2079-2093.

83. El Shamy, T., et al., *The impact of uterine artery embolization on ovarian reserve: A systematic review and meta-analysis.* ACTA OBSTETRICIA ET GYNECOLOGICA SCANDINAVICA, 2020. **99**(1): p. 16-23.

84. Elhenawy, A.M., et al., *Role of preoperative intravenous iron therapy to correct anemia before major surgery: a systematic review and meta-analysis.* SYSTEMATIC REVIEWS, 2021. **10**(1).

85. Eslami, S., et al., *Effectiveness of IT-based interventions on self-management in adult kidney transplant recipients: a systematic review.* BMC MEDICAL INFORMATICS AND DECISION MAKING, 2021. **21**(1).

86. Fasugba, O., et al., *Increased fluid intake for the prevention of urinary tract infection in adults and children in all settings: a systematic review.* JOURNAL OF HOSPITAL INFECTION, 2020. **104**(1): p. 68-77.

87. Fatima, N., et al., *Structural Allograft versus Polyetheretherketone Implants in Patients Undergoing Spinal Fusion Surgery: A Systematic Review and Meta-Analysis.* WORLD NEUROSURGERY, 2020. **136**: p. 101-109.

88. Feng, D.C., et al., *Generating comprehensive comparative evidence on various interventions for penile rehabilitation in patients with erectile dysfunction after radical prostatectomy: a systematic review and network meta-analysis.* TRANSLATIONAL ANDROLOGY AND UROLOGY, 2021. **10**(1): p. 109-124.

89. Feng, F.C., et al., *Xiao-ai-ping injection adjunct with platinum-based chemotherapy for advanced non-small-cell lung cancer: a systematic review and meta-analysis.* BMC COMPLEMENTARY MEDICINE AND THERAPIES, 2020. **20**(1).

90. Feo, C.F., et al., *Laparoscopic versus Open Transverse-Incision Approach for Right Hemicolectomy: A Systematic Review and Meta-Analysis.* MEDICINA-LITHUANIA, 2021. **57**(1).

91. Field, M.S., P. Mithra, and J.P. Pena-Rosas, *Wheat flour fortification with iron and other micronutrients for reducing anaemia and improving iron status in populations.* COCHRANE DATABASE OF SYSTEMATIC REVIEWS, 2021(1).

92. Fleeman, N., et al., *Lenvatinib and sorafenib for differentiated thyroid cancer after radioactive iodine: a systematic review and economic evaluation.* HEALTH TECHNOLOGY ASSESSMENT, 2020. **24**(2): p. 1-+.

93. Fletcher, A.N., et al., *Systematic Review of Subtalar Distraction Arthrodesis for the Treatment of Subtalar Arthritis.* FOOT & ANKLE INTERNATIONAL, 2020. **41**(4): p. 437-448.

94. Formica, V., et al., *A systematic review and meta-analysis of PD-1/PD-L1 inhibitors in specific patient subgroups with advanced gastro-oesophageal junction and gastric adenocarcinoma.* CRITICAL REVIEWS IN ONCOLOGY HEMATOLOGY, 2021. **157**.

95. Forte, A., et al., *The Role of New Technologies to Prevent Suicide in Adolescence: A Systematic Review of the Literature.* MEDICINA-LITHUANIA, 2021. **57**(2).

96. Freixo, C., et al., *Efficacy and safety of sirolimus in the treatment of vascular anomalies: A systematic review.* JOURNAL OF VASCULAR SURGERY, 2020. **71**(1): p. 318-327.

97. Frohlich, T.T., R.D. Rocha, and G. Botton, *Does previous application of silver diammine fluoride influence the bond strength of glass ionomer cement and adhesive systems to dentin? Systematic review and meta-analysis.* INTERNATIONAL JOURNAL OF PAEDIATRIC DENTISTRY, 2020. **30**(1): p. 85-95.

98. Fu, L.L., et al., *Patient-reported outcome measures of edentulous patients restored with single-implant mandibular overdentures: A systematic review.* JOURNAL OF ORAL REHABILITATION, 2021. **48**(1): p. 81-94.

99. Garg, A., et al., *Drug-eluting Stents Versus Bare-metal Stents for Saphenous Vein Graft Interventions: A Systematic Review and Meta-analysis of Studies With Longer Follow-up.* CURRENT PROBLEMS IN CARDIOLOGY, 2021. **46**(1).

100. Garland, E.L., et al., *Mind-Body Therapies for Opioid-Treated Pain A Systematic Review and Meta-analysis.* JAMA INTERNAL MEDICINE, 2020. **180**(1): p. 91-105.

101. Garthwaite, H., J. Stewart, and S. Wilkes, *Multiple pregnancy rate in patients undergoing treatment with clomifene citrate for WHO group II ovulatory disorders: a systematic review.* HUMAN FERTILITY.

102. Gebrie, D., D. Getnet, and T. Manyazewal, *Cardiovascular safety and efficacy of metformin-SGLT2i versus metformin-sulfonylureas in type 2 diabetes: systematic review and meta-analysis of randomized controlled trials.* SCIENTIFIC REPORTS, 2021. **11**(1).

103. Glaser, J., et al., *Interventions to Improve Patient Comprehension in Informed Consent for Medical and Surgical Procedures: An Updated Systematic Review.* MEDICAL DECISION MAKING, 2020. **40**(2): p. 119-143.

104. Gomes, F., et al., *Interventions to increase adherence to micronutrient supplementation during pregnancy: a systematic review.* ANNALS OF THE NEW YORK ACADEMY OF SCIENCES, 2021. **1493**(1): p. 41-58.

105. Goncalves, C.A.M., et al., *Effect of Acute and Chronic Aerobic Exercise on Immunological Markers: A Systematic Review.* FRONTIERS IN PHYSIOLOGY, 2020. **10**.

106. Gong, X.J., et al., *Pregnancy outcomes in female patients exposed to cyclosporin-based versus tacrolimus-based immunosuppressive regimens after liver/kidney transplantation: A systematic review and meta-analysis.* JOURNAL OF CLINICAL PHARMACY AND THERAPEUTICS, 2021. **46**(3): p. 744-753.

107. Gong, Y.N., et al., *Only Tumors Angiographically Identified as Hypervascular Exhibit Lower Intraoperative Blood Loss Upon Selective Preoperative Embolization of Spinal Metastases: Systematic Review and Meta-Analysis.* FRONTIERS IN ONCOLOGY, 2021. **10**.

108. Gottlieb, M., S. Dyer, and G.D. Peksa, *Beta-blockade for the treatment of cardiac arrest due to ventricular fibrillation or pulseless ventricular tachycardia: A systematic review and meta-analysis.* RESUSCITATION, 2020. **146**: p. 118-125.

109. Goya, M., et al., *The use of intracardiac echocardiography catheters in endocardial ablation of cardiac arrhythmia: Meta-analysis of efficiency, effectiveness, and safety outcomes.* JOURNAL OF CARDIOVASCULAR ELECTROPHYSIOLOGY, 2020. **31**(3): p. 664-673.

110. Grenvik, J.M., et al., *Birthing ball for reducing labor pain: a systematic review and meta-analysis of randomized controlled trials.* JOURNAL OF MATERNAL-FETAL & NEONATAL MEDICINE.

111. Grigor, E.J.M., et al., *Interventions to prevent anastomotic leak after esophageal surgery: a systematic review and meta-analysis.* BMC SURGERY, 2021. **21**(1).

112. Gryziak, M., et al., *Milestones in the treatment of hepatocellular carcinoma: A systematic review.* CRITICAL REVIEWS IN ONCOLOGY HEMATOLOGY, 2021. **157**.

113. Guaiana, G., et al., *A Systematic Review of the Use of Telepsychiatry in Depression.* COMMUNITY MENTAL HEALTH JOURNAL, 2021. **57**(1): p. 93-100.

114. Guo, P.P., et al., *Mind-body interventions on stress management in pregnant women: A systematic review and meta-analysis of randomized controlled trials.* JOURNAL OF ADVANCED NURSING, 2021. **77**(1): p. 125-146.

115. Guo, Y.Y., et al., *Comparison of pathologic outcomes of robotic and open resections for rectal cancer: A systematic review and meta-analysis.* PLOS ONE, 2021. **16**(1).

116. Halpin, E., H. Inch, and M. O'Neill, *Dexmedetomidine's Relationship to Delirium in Patients Undergoing Cardiac Surgery A Systematic Review.* CRITICAL CARE NURSING QUARTERLY, 2020. **43**(1): p. 28-38.

117. Hao, J.T., et al., *Efficacy and safety of PARP inhibitors in the treatment of advanced ovarian cancer: An updated systematic review and meta-analysis of randomized controlled trials.* CRITICAL REVIEWS IN ONCOLOGY HEMATOLOGY, 2021. **157**.

118. He, L.Z., et al., *Effectiveness of Postoperative Adjuvant Radiotherapy in Atypical Meningioma Patients After Gross Total Resection: A Meta-Analysis Study.* FRONTIERS IN ONCOLOGY, 2021. **10**.

119. Hermida, R.C., et al., *Extent of asleep blood pressure reduction by hypertension medications is ingestion-time dependent: Systematic review and meta-analysis of published human trials.* SLEEP MEDICINE REVIEWS, 2021. **59**.

120. Hernandez-Vaquero, D., et al., *Survival After Thoracoscopic Surgery or Open Lobectomy: Systematic Review and Meta-Analysis.* ANNALS OF THORACIC SURGERY, 2021. **111**(1): p. 302-313.

121. Hett, D., et al., *Repetitive Transcranial Magnetic Stimulation (rTMS) for the Treatment of Depression in Adolescence: A Systematic Review.* JOURNAL OF AFFECTIVE DISORDERS, 2021. **278**: p. 460-469.

122. Hill, N.R., et al., *A Systematic Review of Network Meta-Analyses and Real-World Evidence Comparing Apixaban and Rivaroxaban in Nonvalvular Atrial Fibrillation.* CLINICAL AND APPLIED THROMBOSIS-HEMOSTASIS, 2020. **26**.

123. Hjelmstrom, P., E.B. Nordbeck, and F. Tiberg, *Optimal dose of buprenorphine in opioid use disorder treatment: a review of pharmacodynamic and efficacy data.* DRUG DEVELOPMENT AND INDUSTRIAL PHARMACY, 2020. **46**(1): p. 1-7.

124. Horta, M., et al., *Chronic oxytocin administration as a tool for investigation and treatment: A cross-disciplinary systematic review.* NEUROSCIENCE AND BIOBEHAVIORAL REVIEWS, 2020. **108**: p. 1-23.

125. Htay, H., et al., *Urgent-startperitoneal dialysis versus haemodialysis for people with chronic kidney disease.* COCHRANE DATABASE OF SYSTEMATIC REVIEWS, 2021(1).

126. Husein-ElAhmed, H. and M. Steinhoff, *Efficacy of topical ivermectin and impact on quality of life in patients with papulopustular rosacea: A systematic review and meta-analysis.* DERMATOLOGIC THERAPY, 2020. **33**(1).

127. Jamil, A., et al., *Spontaneous coronary artery dissection managed with a conservative or revascularization approach: a meta-analysis.* JOURNAL OF CARDIOVASCULAR MEDICINE, 2020. **21**(1): p. 42-50.

128. Jaruvongvanich, V., et al., *Long-term outcomes of Roux-en-Y gastric diversion after failed surgical fundoplication in a large cohort and a systematic review.* SURGERY FOR OBESITY AND RELATED DISEASES, 2021. **17**(1): p. 161-169.

129. Jiang, D.Q., et al., *Rasagiline combined with levodopa therapy versus levodopa monotherapy for patients with Parkinson's disease: a systematic review.* NEUROLOGICAL SCIENCES, 2020. **41**(1): p. 101-109.

130. Jiang, Y., et al., *Efficacy and safety of catheter ablation combined with left atrial appendage occlusion for nonvalvular atrial fibrillation: A systematic review and meta-analysis.* PACE-PACING AND CLINICAL ELECTROPHYSIOLOGY, 2020. **43**(1): p. 123-132.

131. Jiang, Y., S. Su, and Y. Borne, *A meta-analysis of the efficacy of HAART on HIV transmission and its impact on sexual risk behaviours among men who have sex with men.* SCIENTIFIC REPORTS, 2020. **10**(1).

132. Junejo, M.H., et al., *Therapeutic options for erosive pustular dermatosis of the scalp: a systematic review.* BRITISH JOURNAL OF DERMATOLOGY, 2021. **184**(1): p. 25-33.

133. Kalytczak, M.M., et al., *The Effect of Sodium Bicarbonate Supplementation on Electromyographic Muscle Activity in Healthy, Physically Active Individuals: A Systematic Review.* JOURNAL OF DIETARY SUPPLEMENTS, 2021. **18**(3): p. 334-350.

134. Kamalinia, S., et al., *Risk of any hypoglycaemia with newer antihyperglycaemic agents in patients with type 2 diabetes: A systematic review and meta-analysis.* Endocrinol Diabetes Metab, 2020. **3**(1): p. e00100.

135. Kantar, R.S., et al., *Hepatic Artery Microvascular Anastomosis in Liver Transplantation: A Systematic Review of the Literature.* ANNALS OF PLASTIC SURGERY, 2021. **86**(1): p. 96-102.

136. Kao, Y.S. and Y. Hsu, *Adjuvant contralateral neck irradiation for oral cavity cancer - A systematic review and meta-analysis.* AMERICAN JOURNAL OF OTOLARYNGOLOGY, 2021. **42**(2).

137. Kashour, Z., et al., *Efficacy of chloroquine or hydroxychloroquine in COVID-19 patients: a systematic review and meta-analysis.* JOURNAL OF ANTIMICROBIAL CHEMOTHERAPY, 2021. **76**(1): p. 30-42.

138. Kavanagh, B.E., et al., *Systematic review and meta-analysis of the role of personality disorder in randomised controlled trials of pharmacological interventions for adults with mood disorders.* JOURNAL OF AFFECTIVE DISORDERS, 2021. **279**: p. 711-721.

139. Keenan, R.T., et al., *The effect of immunomodulators on the efficacy and tolerability of pegloticase: a systematic review.* SEMINARS IN ARTHRITIS AND RHEUMATISM, 2021. **51**(2): p. 347-352.

140. Kennedy, A.J., et al., *Factors Associated with Long-Term Retention in Buprenorphine-Based Addiction Treatment Programs: a Systematic Review.* JOURNAL OF GENERAL INTERNAL MEDICINE, 2022. **37**(2): p. 332-340.

141. Khaing, K. and B.R. Nair, *Melatonin for delirium prevention in hospitalized patients: A systematic review and meta-analysis.* JOURNAL OF PSYCHIATRIC RESEARCH, 2021. **133**: p. 181-190.

142. Khan, R., et al., *The therapeutic role of Cannabidiol in mental health: a systematic review.* J Cannabis Res, 2020. **2**(1): p. 2.

143. Khaw, S.C., R.A. Anderson, and M.W. Lui, *Systematic review of pregnancy outcomes after fertility-preserving treatment of uterine fibroids.* REPRODUCTIVE BIOMEDICINE ONLINE, 2020. **40**(3): p. 429-444.

144. Kim, D., et al., *Systematic review of randomized controlled trials for chronic fatigue syndrome/myalgic encephalomyelitis (CFS/ME).* JOURNAL OF TRANSLATIONAL MEDICINE, 2020. **18**(1).

145. Kim, S.H., et al., *Effectiveness of quadratus lumborum block for postoperative pain: a systematic review and meta-analysis.* MINERVA ANESTESIOLOGICA, 2020. **86**(5): p. 554-564.

146. Klein, A., et al., *The benefits of mindfulness-based interventions on burnout among health professionals: A systematic review.* EXPLORE-THE JOURNAL OF SCIENCE AND HEALING, 2020. **16**(1): p. 35-43.

147. Koulouris, A.I., et al., *Endoscopic ultrasound-guided celiac plexus neurolysis (EUS-CPN) technique and analgesic efficacy in patients with pancreatic cancer: A systematic review and meta-analysis.* PANCREATOLOGY, 2021. **21**(2): p. 434-442.

148. Kraus, S.W., R. Etuk, and M.N. Potenza, *Current pharmacotherapy for gambling disorder: a systematic review.* EXPERT OPINION ON PHARMACOTHERAPY, 2020. **21**(3): p. 287-296.

149. Kruszecki, C., et al., *A systematic review of integrative medicine for opioid withdrawal.* JOURNAL OF SUBSTANCE ABUSE TREATMENT, 2021. **125**.

150. Kruyt, I.J., et al., *The efficacy of bone-anchored hearing implant surgery in children: A systematic review.* INTERNATIONAL JOURNAL OF PEDIATRIC OTORHINOLARYNGOLOGY, 2020. **132**.

151. Kuszmaul, A.K., E.C. Palmer, and E.K. Frederick, *Lofexidine versus clonidine for mitigation of opioid withdrawal symptoms: A systematic review.* JOURNAL OF THE AMERICAN PHARMACISTS ASSOCIATION, 2020. **60**(1): p. 145-152.

152. Kwon, C.Y., et al., *Non-pharmacological Treatment for Elderly Individuals With Insomnia: A Systematic Review and Network Meta-Analysis.* FRONTIERS IN PSYCHIATRY, 2021. **11**.

153. La Touche, R., et al., *Is aerobic exercise helpful in patients with migraine? A systematic review and meta-analysis.* SCANDINAVIAN JOURNAL OF MEDICINE & SCIENCE IN SPORTS, 2020. **30**(6): p. 965-982.

154. Lagowska, K. and J. Bajerska, *Effects of probiotic supplementation on respiratory infection and immune function in athletes: systematic review and meta-analysis of randomized controlled trials.* J Athl Train, 2021.

155. Lam, P.P.Y., et al., *EFFECTIVENESS OF PIT AND FISSURE SEALANTS FOR PREVENTING AND ARRESTING OCCLUSAL CARIES IN PRIMARY MOLARS: A SYSTEMATIC REVIEW AND META-ANALYSIS.* JOURNAL OF EVIDENCE-BASED DENTAL PRACTICE, 2020. **20**(2).

156. Lan, T.X., et al., *The Association Between Effectiveness of Tinnitus Intervention and Cognitive Function-A Systematic Review.* FRONTIERS IN PSYCHOLOGY, 2021. **11**.

157. Larivee, N. and C.J. Chin, *Aspirin desensitization therapy in aspirin-exacerbated respiratory disease: a systematic review.* INTERNATIONAL FORUM OF ALLERGY & RHINOLOGY, 2020. **10**(4): p. 450-464.

158. Lateef, N., et al., *Percutaneous coronary intervention for chronic total occlusion in patients aged < 75 years versus >= 75 years: a systematic review.* JOURNAL OF COMMUNITY HOSPITAL INTERNAL MEDICINE PERSPECTIVES, 2020. **10**(1): p. 25-31.

159. Lazo-Porras, M., et al., *World Health Organization (WHO) antibiotic regimen against other regimens for the treatment of leprosy: a systematic review and meta-analysis.* BMC INFECTIOUS DISEASES, 2020. **20**(1).

160. Lee, H.S. and J. Lee, *Effects of Combined Exercise and Low Carbohydrate Ketogenic Diet Interventions on Waist Circumference and Triglycerides in Overweight and Obese Individuals: A Systematic Review and Meta-Analysis.* INTERNATIONAL JOURNAL OF ENVIRONMENTAL RESEARCH AND PUBLIC HEALTH, 2021. **18**(2).

161. Lee, H.W., et al., *Renin-angiotensin system blocker and outcomes of COVID-19: a systematic review and meta-analysis.* THORAX, 2021. **76**(5): p. 479-486.

162. Lee, W.C., et al., *Long-term maxillary three dimensional changes following maxillary protraction with or without expansion: A systematic review and meta-analysis.* JOURNAL OF DENTAL SCIENCES, 2021. **16**(1): p. 168-177.

163. Lee, Y., et al., *Laparoscopic Sleeve Gastrectomy Versus Laparoscopic Roux-en-Y Gastric Bypass A Systematic Review and Meta-analysis of Weight Loss, Comorbidities, and Biochemical Outcomes From Randomized Controlled Trials.* ANNALS OF SURGERY, 2021. **273**(1): p. 66-74.

164. Lei, Y.X., et al., *The effects of oral treatment for systemic sclerosis related pulmonary arterial hypertension: A systematic review and meta-analysis.* MODERN RHEUMATOLOGY, 2021. **31**(1): p. 151-161.

165. Lelijveld, N., et al., *Systematic review of the treatment of moderate acute malnutrition using food products.* MATERNAL AND CHILD NUTRITION, 2020. **16**(1).

166. Leow, H.W., E.L. Tan, and M. Black, *Reported outcomes for planned caesarean section versus planned vaginal delivery: A systematic review.* EUROPEAN JOURNAL OF OBSTETRICS & GYNECOLOGY AND REPRODUCTIVE BIOLOGY, 2021. **256**: p. 101-108.

167. Lewis, K., et al., *The efficacy and safety of hydroxychloroquine for COVID-19 prophylaxis: A systematic review and meta-analysis of randomized trials.* PLOS ONE, 2021. **16**(1).

168. Lewis, K., et al., *Safety and Efficacy of Dexmedetomidine in Acutely Ill Adults Requiring Noninvasive Ventilation A Systematic Review and Meta-analysis of Randomized Trials.* CHEST, 2021. **159**(6): p. 2274-2288.

169. Li, F., et al., *Three-dimensional image-guided combined intracavitary and interstitial high-dose-rate brachytherapy in cervical cancer: A systematic review.* BRACHYTHERAPY, 2021. **20**(1): p. 85-94.

170. Li, S.Y., et al., *Analgesic Impact of Single-Shot Versus Continuous Femoral Nerve Block After Total Knee Arthroplasty: A Systematic Review and Meta-Analysis.* ADVANCES IN THERAPY, 2020. **37**(2): p. 671-685.

171. Li, X.F., et al., *Endovascular treatment for ischemic stroke beyond the time window: A meta-analysis.* ACTA NEUROLOGICA SCANDINAVICA, 2020. **141**(1): p. 3-13.

172. Li, X.M., et al., *Timing of renal replacement therapy initiation for acute kidney injury in critically ill patients: a systematic review of randomized clinical trials with meta-analysis and trial sequential analysis.* CRITICAL CARE, 2021. **25**(1).

173. Liang, Z.J., et al., *Efficacy and safety of traditional Chinese medicines for non-alcoholic fatty liver disease: a systematic literature review of randomized controlled trials.* CHINESE MEDICINE, 2021. **16**(1).

174. Lichtenberg, M.K.W., et al., *Endovascular mechanical thrombectomy versus thrombolysis in patients with iliofemoral deep vein thrombosis - a systematic review and meta-analysis.* VASA-EUROPEAN JOURNAL OF VASCULAR MEDICINE, 2021. **50**(1): p. 59-67.

175. Lin, F., et al., *Atherectomy plus drug-coated balloon versus drug-coated balloon only for treatment of femoropopliteal artery lesions: A systematic review and meta-analysis.* VASCULAR, 2021. **29**(6): p. 883-896.

176. Lin, W.S., et al., *Administration of mesenchymal stem cells in diabetic kidney disease: a systematic review and meta-analysis.* STEM CELL RESEARCH & THERAPY, 2021. **12**(1).

177. Litowski, M.L., et al., *Surgical outcomes and complications following distal biceps tendon reconstruction: a systematic review and meta-analysis.* JSES Int, 2021. **5**(1): p. 24-30.

178. Litta, F., et al., *Simple fistula-in-ano: is it all simple? A systematic review.* TECHNIQUES IN COLOPROCTOLOGY, 2021. **25**(4): p. 385-399.

179. Liu, S., et al., *Effectiveness of ultra-long protocol on in vitro fertilization/intracytoplasmic sperm injection-embryo transfer outcome in infertile women with endometriosis: A systematic review and meta-analysis of randomized controlled trials.* JOURNAL OF OBSTETRICS AND GYNAECOLOGY RESEARCH, 2021. **47**(4): p. 1232-1242.

180. Liu, Y.C., I.L. Li, and F.H. Hsiao, *Effectiveness of mindfulness-based intervention on psychotic symptoms for patients with schizophrenia: A meta-analysis of randomized controlled trials.* JOURNAL OF ADVANCED NURSING, 2021. **77**(6): p. 2565-2580.

181. Liu, Y.X., et al., *Efficacy and safety of tigecycline for complicated urinary tract infection: a systematic review.* TRANSLATIONAL ANDROLOGY AND UROLOGY, 2021. **10**(1).

182. Lloyd, D.M., et al., *Is Transcranial Direct Current Stimulation (tDCS) Effective for the Treatment of Pain in Fibromyalgia? A Systematic Review and Meta-Analysis.* JOURNAL OF PAIN, 2020. **21**(11-12): p. 1085-1100.

183. Lo, K.B., et al., *The Effects of SGLT2 Inhibitors on Cardiovascular and Renal Outcomes in Diabetic Patients: A Systematic Review and Meta-Analysis.* CARDIORENAL MEDICINE, 2020. **10**(1): p. 1-10.

184. Lobb, H.S., et al., *Suture rectopexy versus ventral mesh rectopexy for complete full-thickness rectal prolapse and intussusception: systematic review and meta-analysis.* BJS OPEN, 2021. **5**(1).

185. Lor, K.Y., et al., *Does pre-operative urodynamics lead to better outcomes in management of urinary incontinence in women? A linked systematic review and meta-analysis.* EUROPEAN JOURNAL OF OBSTETRICS & GYNECOLOGY AND REPRODUCTIVE BIOLOGY, 2020. **244**: p. 141-153.

186. Louwers, A., et al., *Effects of upper extremity surgery on activities and participation of children with cerebral palsy: a systematic review.* DEVELOPMENTAL MEDICINE AND CHILD NEUROLOGY, 2020. **62**(1): p. 21-27.

187. Lu, Z.X., F. Wang, and H.T. Lv, *Efficacy of infliximab in the treatment of Kawasaki disease: A systematic review and meta-analysis.* EXPERIMENTAL AND THERAPEUTIC MEDICINE, 2021. **21**(1).

188. Lussier, M.E., et al., *The impact of community pharmacist involvement on transitions of care: A systematic review and meta-analysis.* JOURNAL OF THE AMERICAN PHARMACISTS ASSOCIATION, 2020. **60**(1): p. 153-+.

189. Lv, J., et al., *The effect of four Immeasurables meditations on depressive symptoms: A systematic review and meta-analysis.* CLINICAL PSYCHOLOGY REVIEW, 2020. **76**.

190. Lyu, D.Y., et al., *Effect of Tai Chi on post-stroke non-motor disorders: a systematic review and meta-analysis of randomized controlled trials.* CLINICAL REHABILITATION, 2021. **35**(1): p. 26-38.

191. Lyu, Y., et al., *Comparison of Three Methods of Gallbladder Drainage for Patients with Acute Cholecystitis Who Are at High Surgical Risk: A Network Meta-Analysis and Systematic Review.* JOURNAL OF LAPAROENDOSCOPIC & ADVANCED SURGICAL TECHNIQUES, 2021. **31**(11): p. 1295-1302.

192. Ma, A.J., et al., *Oropharyngeal colostrum therapy reduces the incidence of ventilator-associated pneumonia in very low birth weight infants: a systematic review and meta-analysis.* PEDIATRIC RESEARCH, 2021. **89**(1): p. 54-62.

193. Ma, X.T., et al., *Immune checkpoint inhibitor (ICI) combination therapy compared to monotherapy in advanced solid cancer: A systematic review.* JOURNAL OF CANCER, 2021. **12**(5): p. 1318-1333.

194. Maheshwer, B., et al., *Contribution of Multimodal Analgesia to Postoperative Pain Outcomes Immediately After Primary Anterior Cruciate Ligament Reconstruction: A Systematic Review and Meta-analysis of Level 1 Randomized Clinical Trials.* AMERICAN JOURNAL OF SPORTS MEDICINE, 2021. **49**(11): p. 3132-3144.

195. Maijers, I., et al., *Oral steroid-sparing effect of high-dose inhaled corticosteroids in asthma.* EUROPEAN RESPIRATORY JOURNAL, 2020. **55**(1).

196. Maisto, M., et al., *Digital Interventions for Psychological Comorbidities in Chronic Diseases-A Systematic Review.* JOURNAL OF PERSONALIZED MEDICINE, 2021. **11**(1).

197. Malik, A., et al., *Endoscopic Therapy in the Management of Patients With Severe Rectal Bleeding Following Transrectal Ultrasound-Guided Prostate Biopsy: A Case-Based Systematic Review.* J Investig Med High Impact Case Rep, 2021. **9**: p. 23247096211013206.

198. Maloney, K.M., et al., *Electronic and other new media technology interventions for HIV care and prevention: a systematic review.* JOURNAL OF THE INTERNATIONAL AIDS SOCIETY, 2020. **23**(1).

199. Maqsood, M.H., et al., *Efficacy of Long-Term Oral Beta-Blocker Therapy in Patients Who Underwent Percutaneous Coronary Intervention for ST-Segment Elevation Myocardial Infarction With Preserved Left Ventricular Ejection Fraction: A Systematic Review and Meta-analysis.* JOURNAL OF CARDIOVASCULAR PHARMACOLOGY, 2021. **77**(1): p. 87-93.

200. Marafini, I., et al., *Respiratory Tract Infections in Inflammatory Bowel Disease Patients Taking Vedolizumab: A Systematic Review and Meta-Analysis of Randomized Controlled Trials.* FRONTIERS IN PHARMACOLOGY, 2021. **11**.

201. Markovina, N., et al., *Efficacy and safety of oral and inhalation commercial beta-glucan products: Systematic review of randomized controlled trials.* CLINICAL NUTRITION, 2020. **39**(1): p. 40-48.

202. Mehdizadeh, A., et al., *Impact of Parent Engagement in Childhood Obesity Prevention Interventions on Anthropometric Indices among Preschool Children: A Systematic Review.* CHILDHOOD OBESITY, 2020. **16**(1): p. 3-19.

203. Mension, E., I. Alonso, and C. Castelo-Branco, *Genitourinary Syndrome of Menopause: Current Treatment Options in Breast Cancer Survivors - Systematic Review.* MATURITAS, 2021. **143**: p. 47-58.

204. Meshkini, F., et al., *The effect of vitamin D supplementation on insulin-like growth factor-1: A systematic review and meta-analysis of randomized controlled trials.* COMPLEMENTARY THERAPIES IN MEDICINE, 2020. **50**.

205. Michelle, L., et al., *Treatments of Periorbital Hyperpigmentation: A Systematic Review.* DERMATOLOGIC SURGERY, 2021. **47**(1): p. 70-74.

206. Miele, A., et al., *Biometric refractive error after cataract and retina surgery: a systematic review and a benchmark proposal.* EYE, 2021. **35**(11): p. 3049-3055.

207. Mirza, H.N., F.N. Mirza, and K.A. Khatri, *Outcomes and adverse effects of ablative vs nonablative lasers for skin resurfacing: A systematic review of 1093 patients.* DERMATOLOGIC THERAPY, 2021. **34**(1).

208. Mistry, K., et al., *Clinical response to antibiotic regimens in lower limb cellulitis: a systematic review.* CLINICAL AND EXPERIMENTAL DERMATOLOGY, 2021. **46**(1): p. 42-49.

209. Mohamed, H., et al., *Tube cecostomy versus appendicostomy for antegrade enemas in the management of fecal incontinence in children: A systematic review.* JOURNAL OF PEDIATRIC SURGERY, 2020. **55**(7): p. 1196-1200.

210. Mohammad, A., et al., *The effects of carnitine supplementation on clinical characteristics of patients with non-alcoholic fatty liver disease: A systematic review and meta-analysis of randomized controlled trials.* COMPLEMENTARY THERAPIES IN MEDICINE, 2020. **48**.

211. Molena, E., E. King, and C. Davies-Husband, *Octreotide versus oral dietary modification for the treatment of chylous fistula following neck dissection: A systematic review and meta-analysis.* CLINICAL OTOLARYNGOLOGY, 2021. **46**(3): p. 474-484.

212. Mongkhon, P., et al., *Non-vitamin K oral anticoagulants and risk of fractures: a systematic review and meta-analysis.* EUROPACE, 2021. **23**(1): p. 39-48.

213. Moore, J.V., et al., *Exocrine Pancreatic Insufficiency After Pancreatectomy for Malignancy: Systematic Review and Optimal Management Recommendations.* JOURNAL OF GASTROINTESTINAL SURGERY, 2021. **25**(9): p. 2317-2327.

214. Muhammad, H., P. Santhanam, and J.O. Russell, *Radiofrequency ablation and thyroid nodules: updated systematic review.* ENDOCRINE, 2021. **72**(3): p. 619-632.

215. Mujahid, N., et al., *Microneedling as a Treatment for Acne Scarring: A Systematic Review.* DERMATOLOGIC SURGERY, 2020. **46**(1): p. 86-92.

216. Mukhtar, N.B., et al., *Effectiveness of Hands-Off Therapy in the Management of Primary Headache: A Systematic Review and Meta-Analysis.* EVALUATION & THE HEALTH PROFESSIONS, 2022. **45**(2): p. 183-203.

217. Mulcahy, M.J., A. Dower, and M. Tait, *Orthosis versus no orthosis for the treatment of thoracolumbar burst fractures: A systematic review.* JOURNAL OF CLINICAL NEUROSCIENCE, 2021. **85**: p. 49-56.

218. Naidoo, A., et al., *Use of Biologics in Pityriasis Rubra Pilaris Refractory to First-Line Systemic Therapy: A Systematic Review.* JOURNAL OF CUTANEOUS MEDICINE AND SURGERY, 2020. **24**(1): p. 73-78.

219. Najafi, M., et al., *The role of melatonin on doxorubicin-induced cardiotoxicity: A systematic review.* LIFE SCIENCES, 2020. **241**.

220. Nakajima, D., et al., *Effectiveness of intravenous lidocaine in preventing postoperative nausea and vomiting in pediatric patients: A systematic review and meta-analysis.* PLOS ONE, 2020. **15**(1).

221. Nakasu, S., et al., *Malignant transformation of WHO grade I meningiomas after surgery or radiosurgery: systematic review and meta-analysis of observational studies.* Neurooncol Adv, 2020. **2**(1): p. vdaa129.

222. Nasiri, M.J., et al., *Antibiotic therapy success rate in pulmonary Mycobacterium avium complex: a systematic review and meta-analysis.* EXPERT REVIEW OF ANTI-INFECTIVE THERAPY, 2020. **18**(3): p. 263-273.

223. Neelakantan, P., et al., *Oral health-related quality of life (OHRQoL) before and after endodontic treatment: a systematic review.* CLINICAL ORAL INVESTIGATIONS, 2020. **24**(1): p. 25-36.

224. Neville, J.J., et al., *Therapeutic strategies for stricturing Crohn's disease in childhood: a systematic review.* PEDIATRIC SURGERY INTERNATIONAL, 2021. **37**(5): p. 569-577.

225. Ni, X.X., et al., *Acupuncture for Radiation-Induced Xerostomia in Cancer Patients: A Systematic Review and Meta-Analysis.* INTEGRATIVE CANCER THERAPIES, 2020. **19**.

226. Ninan, K., et al., *Y Neonatal and Maternal Outcomes of Lower Versus Standard Doses of Antenatal Corticosteroids for Women at Risk of Preterm Delivery: A Systematic Review of Randomized Controlled Trials.* JOURNAL OF OBSTETRICS AND GYNAECOLOGY CANADA, 2021. **43**(1): p. 74-81.

227. Niu, H.L. and J.Y. Xiao, *The efficacy and safety of probiotics in patients with irritable bowel syndrome: Evidence based on 35 randomized controlled trials.* INTERNATIONAL JOURNAL OF SURGERY, 2020. **75**: p. 116-127.

228. Nwadiugwu, M.C., *Inflammatory Activities in Type 2 Diabetes Patients With Co-morbid Angiopathies and Exploring Beneficial Interventions: A Systematic Review.* FRONTIERS IN PUBLIC HEALTH, 2021. **8**.

229. O'Callaghan, K.M., et al., *Vitamin D in Breastfed Infants: Systematic Review of Alternatives to Daily Supplementation.* ADVANCES IN NUTRITION, 2020. **11**(1): p. 144-159.

230. Ochoa-Urrea, M., et al., *Electrical Stimulation-Induced Seizures and Breathing Dysfunction: A Systematic Review of New Insights Into the Epileptogenic and Symptomatogenic Zones.* FRONTIERS IN HUMAN NEUROSCIENCE, 2021. **14**.

231. Odegaard, N.B., et al., *Digital learning designs in physiotherapy education: a systematic review and meta-analysis.* BMC MEDICAL EDUCATION, 2021. **21**(1).

232. Ohlsson, A. and J.B. Lacy, *Intravenous immunoglobulin for preventing infection in preterm and/or low birth weight infants.* COCHRANE DATABASE OF SYSTEMATIC REVIEWS, 2020(1).

233. Oosterhoff, T.C.H., et al., *Laser treatment of specific scar characteristics in hypertrophic scars and keloid: A systematic review.* JOURNAL OF PLASTIC RECONSTRUCTIVE AND AESTHETIC SURGERY, 2021. **74**(1): p. 48-64.

234. Pan, Y.Q., et al., *Acupuncture for Hormone Therapy-Related Side Effects in Breast Cancer Patients: A GRADE-Assessed Systematic Review and Updated Meta-Analysis.* INTEGRATIVE CANCER THERAPIES, 2020. **19**.

235. Pandey, A.K., et al., *Lower versus Standard INR Targets in Atrial Fibrillation: A Systematic Review and Meta-Analysis of Randomized Controlled Trials.* THROMBOSIS AND HAEMOSTASIS, 2020. **120**(3): p. 484-494.

236. Pardos-Gascon, E.M., et al., *Differential efficacy between cognitive-behavioral therapy and mindfulness-based therapies for chronic pain: Systematic review.* INTERNATIONAL JOURNAL OF CLINICAL AND HEALTH PSYCHOLOGY, 2021. **21**(1).

237. Parrondo, R.D., et al., *Efficacy of proteasome inhibitor-based maintenance following autologous transplantation in multiple myeloma: A systematic review and meta-analysis.* EUROPEAN JOURNAL OF HAEMATOLOGY, 2021. **106**(1): p. 40-48.

238. Pathirana, M.M., et al., *Cardiovascular risk factors in offspring exposed to gestational diabetes mellitus in utero: systematic review and meta-analysis.* JOURNAL OF DEVELOPMENTAL ORIGINS OF HEALTH AND DISEASE, 2020. **11**(6): p. 599-616.

239. Patini, R., et al., *The Effect of Different Antibiotic Regimens on Bacterial Resistance: A Systematic Review.* ANTIBIOTICS-BASEL, 2020. **9**(1).

240. Pazan, F., et al., *Current evidence on the impact of medication optimization or pharmacological interventions on frailty or aspects of frailty: a systematic review of randomized controlled trials.* EUROPEAN JOURNAL OF CLINICAL PHARMACOLOGY, 2021. **77**(1): p. 1-12.

241. Peixoto, K.O., et al., *Temporomandibular disorders and the use of traditional and laser acupuncture: a systematic review.* CRANIO-THE JOURNAL OF CRANIOMANDIBULAR & SLEEP PRACTICE.

242. Pilger, T.L., D.F. Francisco, and F.J.C. dos Reis, *Effect of sentinel lymph node biopsy on upper limb function in women with early breast cancer: A systematic review of clinical trials.* EJSO, 2021. **47**(7): p. 1497-1506.

243. Piski, Z., et al., *TNF-Alpha Inhibitors and Rhinosinusitis-A Systematic Review and Meta-Analysis.* AMERICAN JOURNAL OF RHINOLOGY & ALLERGY, 2020. **34**(3): p. 436-442.

244. Pitsillides, A., D. Stasinopoulos, and K. Giannakou, *The effects of cognitive behavioural therapy delivered by physical therapists in knee osteoarthritis pain: A systematic review and meta-analysis of randomized controlled trials.* JOURNAL OF BODYWORK AND MOVEMENT THERAPIES, 2021. **25**: p. 157-164.

245. Poonai, N., et al., *Intranasal Dexmedetomidine for Procedural Distress in Children: A Systematic Review.* PEDIATRICS, 2020. **145**(1).

246. Pourmand, A., et al., *Topical capsaicin for the treatment of cannabinoid hyperemesis syndrome, a systematic review and meta-analysis.* AMERICAN JOURNAL OF EMERGENCY MEDICINE, 2021. **43**: p. 35-40.

247. Pourmasoumi, M., et al., *Effect of pycnogenol supplementation on blood pressure: A systematic review and meta-analysis of clinical trials.* PHYTOTHERAPY RESEARCH, 2020. **34**(1): p. 67-76.

248. Pranata, R., et al., *Minimal Invasive Surgery Instrumented Fusion versus Conventional Open Surgical Instrumented Fusion for the Treatment of Spinal Metastases: A Systematic Review and Meta-analysis.* WORLD NEUROSURGERY, 2021. **148**: p. E264-E274.

249. Pranata, R., et al., *Remote ischemic preconditioning reduces the incidence of contrast-induced nephropathy in patients undergoing coronary angiography/intervention: Systematic review and meta-analysis of randomized controlled trials.* CATHETERIZATION AND CARDIOVASCULAR INTERVENTIONS, 2020. **96**(6): p. 1200-1212.

250. Premat, K., et al., *Rescue stenting versus medical care alone in refractory large vessel occlusions: a systematic review and meta-analysis.* NEURORADIOLOGY, 2020. **62**(5): p. 629-637.

251. Qi, M.L., et al., *Tai Chi Combined With Resistance Training for Adults Aged 50 Years and Older: A Systematic Review.* JOURNAL OF GERIATRIC PHYSICAL THERAPY, 2020. **43**(1): p. 32-41.

252. Qu, H.C., et al., *Efficacy and Safety of Chemotherapy Regimens in Advanced or Metastatic Bladder and Urothelial Carcinomas: An Updated Network Meta-Analysis.* FRONTIERS IN PHARMACOLOGY, 2020. **10**.

253. Queiroga, T.L.O., et al., *Mitomycin C in the Endoscopic Treatment of Laryngotracheal Stenosis: Systematic Review and Proportional Meta-Analysis.* Int Arch Otorhinolaryngol, 2020. **24**(1): p. e112-e124.

254. Raban, M.Z., et al., *Effectiveness of interventions targeting antibiotic use in long-term aged care facilities: a systematic review and meta-analysis.* BMJ OPEN, 2020. **10**(1).

255. Rahmani, J., et al., *The effect of Saffron supplementation on waist circumference, HA1C, and glucose metabolism: A systematic review and meta-analysis of randomized clinical trials.* COMPLEMENTARY THERAPIES IN MEDICINE, 2020. **49**.

256. Ramirez-Velez, R., et al., *Effects of Exercise Interventions on Inflammatory Parameters in Acutely Hospitalized Older Patients: A Systematic Review and Meta-Analysis of Randomized Controlled Trials.* JOURNAL OF CLINICAL MEDICINE, 2021. **10**(2).

257. Rashid, A.A., et al., *Effectiveness of pretend medical play in improving children's health outcomes and well-being: a systematic review.* BMJ OPEN, 2021. **11**(1).

258. Rasmussen, L.F., et al., *Impact of transitional care interventions on hospital readmissions in older medical patients: a systematic review.* BMJ OPEN, 2021. **11**(1).

259. Rayce, S.B., et al., *Effects of parenting interventions for mothers with depressive symptoms and an infant: systematic review and meta-analysis.* BJPSYCH OPEN, 2020. **6**(1).

260. Reid, G.A., et al., *FIRST FAILED MACULAR HOLE SURGERY OR REOPENING OF A PREVIOUSLY CLOSED HOLE Do We Gain by Reoperating?-A Systematic Review and Meta-analysis.* RETINA-THE JOURNAL OF RETINAL AND VITREOUS DISEASES, 2020. **40**(1): p. 1-15.

261. Rekhi, U., R.Q. Catunda, and M.P. Gibson, *Surgically accelerated orthodontic techniques and periodontal response: a systematic review.* EUROPEAN JOURNAL OF ORTHODONTICS, 2020. **42**(6): p. 635-642.

262. Ren, J.Y., et al., *Effect of proanthocyanidins on blood pressure: A systematic review and meta-analysis of randomized controlled trials.* PHARMACOLOGICAL RESEARCH, 2021. **165**.

263. Romoli, M., et al., *Switching between direct oral anticoagulants: a systematic review and meta-analysis.* JOURNAL OF THROMBOSIS AND THROMBOLYSIS, 2021. **52**(2): p. 560-566.

264. Roodenrijs, N.M.T., et al., *Pharmacological and non-pharmacological therapeutic strategies in difficult-to-treat rheumatoid arthritis: a systematic literature review informing the EULAR recommendations for the management of difficult-to-treat rheumatoid arthritis.* RMD OPEN, 2021. **7**(1).

265. Ross, J.M., et al., *Isoniazid preventive therapy plus antiretroviral therapy for the prevention of tuberculosis: a systematic review and meta-analysis of individual participant data.* Lancet HIV, 2021. **8**(1): p. e8-e15.

266. Roy, M., et al., *Removal of osseointegrated dental implants: a systematic review of explantation techniques.* CLINICAL ORAL INVESTIGATIONS, 2020. **24**(1): p. 47-60.

267. Sabe, M., S. Kaiser, and O. Sentissi, *Physical exercise for negative symptoms of schizophrenia: Systematic review of randomized controlled trials and meta-analysis.* GENERAL HOSPITAL PSYCHIATRY, 2020. **62**: p. 13-20.

268. Samadi, M., et al., *The role of taurine on chemotherapy-induced cardiotoxicity: A systematic review of non-clinical study.* LIFE SCIENCES, 2021. **265**.

269. Samy, A., et al., *Perioperative nonhormonal pharmacological interventions for bleeding reduction during open and minimally invasive myomectomy: a systematic review and network meta-analysis.* FERTILITY AND STERILITY, 2020. **113**(1): p. 224-+.

270. Sartori, N.S., N.P.B. de Andrade, and R.M.D. Chakr, *Incidence of tuberculosis in patients receiving anti-TNF therapy for rheumatic diseases: a systematic review.* CLINICAL RHEUMATOLOGY, 2020. **39**(5): p. 1439-1447.

271. Scherer, S., et al., *Changes in health-related outcomes among colorectal cancer patients undergoing inpatient rehabilitation therapy: a systematic review of observational and interventional studies.* ACTA ONCOLOGICA, 2021. **60**(1): p. 124-134.

272. Schild, S.D., et al., *Surgical Management of Sialorrhea: A Systematic Review and Meta-analysis.* OTOLARYNGOLOGY-HEAD AND NECK SURGERY, 2021. **165**(4): p. 507-518.

273. Schimmack, S., et al., *Meta-analysis of alpha-blockade versus no blockade before adrenalectomy for phaeochromocytoma.* BRITISH JOURNAL OF SURGERY, 2020. **107**(2): p. E102-E108.

274. Schmidt, R., et al., *Safety and efficacy of baroreflex activation therapy for heart failure with reduced ejection fraction: a rapid systematic review.* ESC HEART FAILURE, 2020. **7**(1): p. 3-14.

275. Schmidt, S., et al., *Intravesical Bacillus Calmette-Guerin versus mitomycin C for Ta and T1 bladder cancer.* COCHRANE DATABASE OF SYSTEMATIC REVIEWS, 2020(1).

276. Schyrr, F., et al., *Perioperative care of children with sickle cell disease: A systematic review and clinical recommendations.* AMERICAN JOURNAL OF HEMATOLOGY, 2020. **95**(1): p. 78-96.

277. Sconza, C., et al., *Oxygen-Ozone Therapy for the Treatment of Knee Osteoarthritis: A Systematic Review of Randomized Controlled Trials.* ARTHROSCOPY-THE JOURNAL OF ARTHROSCOPIC AND RELATED SURGERY, 2020. **36**(1): p. 277-286.

278. Severo, J.S., et al., *Effects of glutamine supplementation on inflammatory bowel disease: A systematic review of clinical trials.* CLINICAL NUTRITION ESPEN, 2021. **42**: p. 53-60.

279. Shaikh, M.S., et al., *Long-term Clinical Performance of Regeneration versus Conservative Surgery in the Treatment of Infra-bony Defects:A systematic review.* J Int Acad Periodontol, 2021. **23**(1): p. 31-56.

280. Singh, A.K. and R. Singh, *Efficacy and safety of lorcaserin in obesity: a systematic review and meta-analysis of randomized controlled trials.* EXPERT REVIEW OF CLINICAL PHARMACOLOGY, 2020. **13**(2): p. 183-190.

281. Skiba, M.B., et al., *Dietary Interventions for Adult Survivors of Adolescent and Young Adult Cancers: A Systematic Review and Narrative Synthesis.* JOURNAL OF ADOLESCENT AND YOUNG ADULT ONCOLOGY, 2020. **9**(3): p. 315-327.

282. Smriti, K., et al., *Intra-lesional medicaments for the management of intra-osseous lesions of maxilla and mandible-systematic review.* JOURNAL OF INTERNATIONAL SOCIETY OF PREVENTIVE AND COMMUNITY DENTISTRY, 2020. **10**(1): p. 36-45.

283. Song, Y., et al., *Current Evidence on Traditional Chinese Exercises for Quality of Life in Patients With Essential Hypertension: A Systematic Review and Meta-Analysis.* FRONTIERS IN CARDIOVASCULAR MEDICINE, 2021. **7**.

284. Sousa, S.J.L., et al., *Early clinical performance of resin cements in glass-ceramic posterior restorations in adult vital teeth: A systematic review and meta-analysis.* JOURNAL OF PROSTHETIC DENTISTRY, 2020. **123**(1): p. 61-70.

285. Stahl, M., et al., *Use of immunosuppressive therapy for management of myelodysplastic syndromes: a systematic review and meta-analysis.* HAEMATOLOGICA, 2020. **105**(1): p. 102-111.

286. Strickland, B.A., et al., *Neuroprotective effect of minocycline against acute brain injury in clinical practice: A systematic review.* JOURNAL OF CLINICAL NEUROSCIENCE, 2021. **86**: p. 50-57.

287. Suarez, S., et al., *Uterine balloon tamponade for the treatment of postpartum hemorrhage: a systematic review and meta-analysis.* AMERICAN JOURNAL OF OBSTETRICS AND GYNECOLOGY, 2020. **222**(4).

288. Sun, L., et al., *Neoadjuvant EGFR-TKI Therapy for EGFR-Mutant NSCLC: A Systematic Review and Pooled Analysis of Five Prospective Clinical Trials.* FRONTIERS IN ONCOLOGY, 2021. **10**.

289. Sun, L., et al., *Feasibility of laparoscopy gastrectomy for gastric cancer in the patients with high body mass index: A systematic review and meta-analysis.* ASIAN JOURNAL OF SURGERY, 2020. **43**(1): p. 69-77.

290. Sun, M.T., et al., *Meta-Analysis of Intraocular Bleeding With Dual Antiplatelet Therapy Using P2Y12 Inhibitors Prasugrel or Ticagrelor.* AMERICAN JOURNAL OF CARDIOLOGY, 2020. **125**(8): p. 1280-1283.

291. Swami, R.K., et al., *Bone replacement grafts with guided tissue regeneration in treatment of grade II furcation defects: a systematic review and meta-analysis.* CLINICAL ORAL INVESTIGATIONS, 2021. **25**(3): p. 807-821.

292. Swords, C.E., et al., *The Use of Postoperative Antibiotics Following Endoscopic Sinus Surgery for Chronic Rhinosinusitis: A Systematic Review and Meta-analysis.* AMERICAN JOURNAL OF RHINOLOGY & ALLERGY, 2021. **35**(5): p. 700-712.

293. Tadount, F., et al., *Is there a difference in the immune response, efficacy, effectiveness and safety of seasonal influenza vaccine in males and females? - A systematic review.* VACCINE, 2020. **38**(3): p. 444-459.

294. Tamura, J.K., et al., *Management of cognitive impairment in bipolar disorder: a systematic review of randomized controlled trials.* CNS Spectr, 2021: p. 1-22.

295. Tan, M.S.A., et al., *A systematic review and meta-analysis of the association between clozapine and norclozapine serum levels and peripheral adverse drug reactions.* PSYCHOPHARMACOLOGY, 2021. **238**(3): p. 615-637.

296. Tang, A.B., et al., *Perioperative and Long-Term Outcomes of Robot-Assisted Partial Nephrectomy: A Systematic Review.* AMERICAN SURGEON, 2021. **87**(1): p. 21-29.

297. Tanzer, T., et al., *Varenicline for cognitive impairment in people with schizophrenia: systematic review and meta-analysis.* PSYCHOPHARMACOLOGY, 2020. **237**(1): p. 11-19.

298. Tassi, A., N. Parisi, and A.P. Londero, *Misoprostol administration prior to intrauterine contraceptive device insertion: a systematic review and meta-analysis of randomised controlled trials.* EUROPEAN JOURNAL OF CONTRACEPTION AND REPRODUCTIVE HEALTH CARE, 2020. **25**(1): p. 76-86.

299. Tejada, S., et al., *Neuraminidase inhibitors are effective and safe in reducing influenza complications: meta-analysis of randomized controlled trials.* EUROPEAN JOURNAL OF INTERNAL MEDICINE, 2021. **86**: p. 54-65.

300. Teo, J.Y.K., R. Turner, and M. Self, *Effect of exercise prehabilitation on functional status of patients undergoing bowel resection: a systematic review.* ANZ JOURNAL OF SURGERY, 2020. **90**(5): p. 693-701.

301. Thapa, R.K., et al., *Effects of Complex Training on Sprint, Jump, and Change of Direction Ability of Soccer Players: A Systematic Review and Meta-Analysis.* FRONTIERS IN PSYCHOLOGY, 2021. **11**.

302. Tian, P.R., et al., *Laparoscopic Proximal Gastrectomy Versus Laparoscopic Total Gastrectomy for Proximal Gastric Cancer: A Systematic Review and Meta-Analysis.* FRONTIERS IN ONCOLOGY, 2021. **10**.

303. Tong, Y.L., et al., *Clinical efficacy and safety of Tanreqing injection combined with antibiotics versus antibiotics alone in the treatment of pulmonary infection patients after chemotherapy with lung cancer: A systematic review and meta-analysis.* PHYTOTHERAPY RESEARCH, 2021. **35**(1): p. 122-137.

304. Tonneau, M., et al., *Stereotactic body radiotherapy for locally advanced pancreatic cancer: A systemic review.* CANCER RADIOTHERAPIE, 2021. **25**(3): p. 283-295.

305. Tutunchi, H., M. Saghafi-Asl, and A. Ostadrahimi, *A systematic review of the effects of oleoylethanolamide, a high-affinity endogenous ligand of PPAR-alpha, on the management and prevention of obesity.* CLINICAL AND EXPERIMENTAL PHARMACOLOGY AND PHYSIOLOGY, 2020. **47**(4): p. 543-552.

306. Tymofiyeva, O. and R. Gaschler, *Training-Induced Neural Plasticity in Youth: A Systematic Review of Structural and Functional MRI Studies.* FRONTIERS IN HUMAN NEUROSCIENCE, 2021. **14**.

307. Uba, R.O., K. Ankoma-Darko, and S.K. Park, *International comparison of mitigation strategies for addressing opioid misuse: A systematic review.* JOURNAL OF THE AMERICAN PHARMACISTS ASSOCIATION, 2020. **60**(1): p. 195-204.

308. Ueshima, D., et al., *Transcatheter aortic valve replacement for bicuspid aortic valve stenosis with first- and new-generation bioprostheses: A systematic review and meta-analysis.* INTERNATIONAL JOURNAL OF CARDIOLOGY, 2020. **298**: p. 76-82.

309. Urban, M.L., et al., *Comparison of treatments for the prevention of fetal growth restriction in obstetric antiphospholipid syndrome: a systematic review and network meta-analysis.* INTERNAL AND EMERGENCY MEDICINE, 2021. **16**(5): p. 1357-1367.

310. van Vuuren, J., et al., *Reshaping healthcare delivery for elderly patients: the role of community paramedicine; a systematic review.* BMC HEALTH SERVICES RESEARCH, 2021. **21**(1).

311. Vania, R., R. Pranata, and S.T. Tan, *Intralesional measles-mumps-rubella is associated with a higher complete response in cutaneous warts: a systematic review and meta-analysis of randomized controlled trial including GRADE qualification.* JOURNAL OF DERMATOLOGICAL TREATMENT, 2021. **32**(8): p. 1010-1017.

312. Varotto, L., et al., *The Closure of Patent Foramen Ovale in Preventing Subsequent Neurological Events: A Bayesian Network Meta-Analysis to Identify the Best Device.* CEREBROVASCULAR DISEASES, 2020. **49**(2): p. 124-134.

313. Vegivinti, C.T.R., et al., *Remdesivir therapy in patients with COVID-19: A systematic review and meta-analysis of randomized controlled trials.* ANNALS OF MEDICINE AND SURGERY, 2021. **62**: p. 43-48.

314. Velando-Soriano, A., et al., *Impact of social support in preventing burnout syndrome in nurses: A systematic review.* JAPAN JOURNAL OF NURSING SCIENCE, 2020. **17**(1).

315. Vis, R., et al., *The effects of pharmacological interventions on quality of life and fatigue in sarcoidosis: a systematic review.* EUROPEAN RESPIRATORY REVIEW, 2020. **29**(155).

316. Walsh, K.A., et al., *The relationship between procedural volume and patient outcomes for percutaneous coronary interventions: a systematic review and meta-analysis.* HRB Open Res, 2021. **4**: p. 10.

317. Wang, A.H., et al., *Effects of sodium-glucose cotransporter 2 inhibitors on risk of venous thromboembolism in patients with type 2 diabetes: A systematic review and meta-analysis.* DIABETES-METABOLISM RESEARCH AND REVIEWS, 2020. **36**(1).

318. Wang, B.C., et al., *Photodynamic therapy with methyl-5-aminolevulinate for basal cell carcinoma: A systematic review and meta-analysis.* PHOTODIAGNOSIS AND PHOTODYNAMIC THERAPY, 2020. **29**.

319. Wang, G.N., et al., *Game-based brain training for improving cognitive function in community-dwelling older adults: A systematic review and meta-regression.* ARCHIVES OF GERONTOLOGY AND GERIATRICS, 2021. **92**.

320. Wang, H., et al., *Hemodialysis and risk of acute pancreatitis: A systematic review and meta-analysis.* PANCREATOLOGY, 2021. **21**(1): p. 89-94.

321. Wang, P., et al., *Efficacy and safety of interleukin-17A inhibitors in patients with ankylosing spondylitis: a systematic review and meta-analysis of randomized controlled trials.* CLINICAL RHEUMATOLOGY, 2021. **40**(8): p. 3053-3065.

322. Wang, W.Y., et al., *Effects of vitamin E supplementation on the risk and progression of AD: a systematic review and meta-analysis.* NUTRITIONAL NEUROSCIENCE, 2021. **24**(1): p. 13-22.

323. Wang, Y.W., et al., *Can Intermittent Pneumatic Compression Reduce the Incidence of Venous Thrombosis in Critically Ill Patients: A Systematic Review and Meta-Analysis.* CLINICAL AND APPLIED THROMBOSIS-HEMOSTASIS, 2020. **26**.

324. Watters, M., et al., *Short versus extended progesterone supplementation for luteal phase support in fresh IVF cycles: a systematic review and meta-analysis.* REPRODUCTIVE BIOMEDICINE ONLINE, 2020. **40**(1): p. 143-150.

325. Williams, Z.F. and E.D. Dillavou, *A systematic review of venous stents for iliac and venacaval occlusive disease.* JOURNAL OF VASCULAR SURGERY-VENOUS AND LYMPHATIC DISORDERS, 2020. **8**(1): p. 145-153.

326. Wouters, Y., et al., *Use of Catheter Lock Solutions in Patients Receiving Home Parenteral Nutrition: A Systematic Review and Individual-Patient Data Meta-analysis.* JOURNAL OF PARENTERAL AND ENTERAL NUTRITION, 2020. **44**(7): p. 1198-1209.

327. Xara-Leite, F., et al., *The cement-in-cement technique is a reliable option in hip arthroplasty revision surgery: a systematic review.* EUROPEAN JOURNAL OF ORTHOPAEDIC SURGERY AND TRAUMATOLOGY, 2021. **31**(1): p. 7-22.

328. Xiao, X., et al., *Effectiveness and Safety of Acupuncture for Perimenopausal Depression: A Systematic Review and Meta-Analysis of Randomized Controlled Trials.* EVIDENCE-BASED COMPLEMENTARY AND ALTERNATIVE MEDICINE, 2020. **2020**.

329. Xie, H.M., et al., *Effectiveness of Botulinum Toxin A in Treatment of Hemiplegic Shoulder Pain: A Systematic Review and Meta-analysis.* ARCHIVES OF PHYSICAL MEDICINE AND REHABILITATION, 2021. **102**(9): p. 1775-1787.

330. Xu, H., et al., *Efficacy and safety of Chinese patent medicine (Jinlong capsule) in the treatment of advanced hepatocellular carcinoma: a meta-analysis.* BIOSCIENCE REPORTS, 2020. **40**.

331. Xu, J., et al., *Transanal drainage tubes vs metallic stents for acute malignant left-sided bowel obstruction A systematic review and meta-analysis.* MEDICINE, 2020. **99**(2).

332. Xu, M.J. and B. Dai, *Inhaled antibiotics therapy for stable non-cystic fibrosis bronchiectasis: a meta-analysis.* THERAPEUTIC ADVANCES IN RESPIRATORY DISEASE, 2020. **14**.

333. Xu, W., et al., *Bronchoscopic lung volume reduction procedures for emphysema: A network meta-analysis.* MEDICINE, 2020. **99**(5).

334. Xu, X.X., et al., *Short versus standard implants for single-crown restorations in the posterior region: A systematic review and meta-analysis.* JOURNAL OF PROSTHETIC DENTISTRY, 2020. **124**(5): p. 530-538.

335. Yamamoto, R., et al., *Efficacy of aerobic exercise on the cardiometabolic and renal outcomes in patients with chronic kidney disease: a systematic review of randomized controlled trials.* JOURNAL OF NEPHROLOGY, 2021. **34**(1): p. 155-164.

336. Yang, H., et al., *The effect of statins on advanced prostate cancer patients with androgen deprivation therapy or abiraterone/enzalutamide: A systematic review and meta-analysis.* JOURNAL OF CLINICAL PHARMACY AND THERAPEUTICS, 2020. **45**(3): p. 488-495.

337. Yang, S.S., et al., *Efficacy and Safety of Guizhi Decoction AssociatedFormulas for Allergic Rhinitis: A Systematic Review.* EVIDENCE-BASED COMPLEMENTARY AND ALTERNATIVE MEDICINE, 2021. **2021**.

338. Yang, S.S., et al., *Effect of Induction Chemotherapy in Nasopharyngeal Carcinoma: An Updated Meta-Analysis.* FRONTIERS IN ONCOLOGY, 2021. **10**.

339. Yausep, O.E., I. Madhi, and D. Trigkilidas, *Platelet rich plasma for treatment of osteochondral lesions of the talus: A systematic review of clinical trials.* JOURNAL OF ORTHOPAEDICS, 2020. **18**: p. 218-225.

340. Yildiz, E., *The effects of acceptance and commitment therapy in psychosis treatment: A systematic review of randomized controlled trials.* PERSPECTIVES IN PSYCHIATRIC CARE, 2020. **56**(1): p. 149-167.

341. Yoshii, T., et al., *Comparison of anterior decompression with fusion and posterior decompression with fusion for cervical spondylotic myelopathy-A systematic review and meta-analysis.* JOURNAL OF ORTHOPAEDIC SCIENCE, 2020. **25**(6): p. 938-945.

342. Yu, C.Q., et al., *Pedicle screw placement in spinal neurosurgery using a 3D-printed drill guide template: a systematic review and meta-analysis.* JOURNAL OF ORTHOPAEDIC SURGERY AND RESEARCH, 2020. **15**(1).

343. Yu, H., et al., *Comparative Effectiveness and Safety of Anterior Cervical Corpectomy with Fusion, Laminoplasty, and Laminectomy and Instrumented Fusion for Ossification of the Posterior Longitudinal Ligament: A Systematic Review and Network Meta-Analysis.* JOURNAL OF INVESTIGATIVE SURGERY, 2022. **35**(3): p. 667-676.

344. Zajac, K., M.K. Ginley, and R. Chang, *Treatments of internet gaming disorder: a systematic review of the evidence.* EXPERT REVIEW OF NEUROTHERAPEUTICS, 2020. **20**(1): p. 85-93.

345. Zeng, J., et al., *Effects of 5-aminosalicylates or thiopurines on the progression of low-grade dysplasia in patients with inflammatory bowel disease: a systematic review and meta-analysis.* INTERNATIONAL JOURNAL OF COLORECTAL DISEASE, 2021. **36**(1): p. 11-18.

346. Zhang, Q., et al., *Cognitive behavioral therapy for depression and anxiety of Parkinson's disease: A systematic review and meta-analysis.* COMPLEMENTARY THERAPIES IN CLINICAL PRACTICE, 2020. **39**.

347. Zhang, Q.Y., et al., *Comparative risk of fracture for bariatric procedures in patients with obesity: A systematic review and Bayesian network meta-analysis.* INTERNATIONAL JOURNAL OF SURGERY, 2020. **75**: p. 13-23.

348. Zhang, Y., et al., *Effect of omega-3 fatty acids supplementation during childhood in preventing allergic disease: a systematic review and Meta-Analysis.* JOURNAL OF ASTHMA, 2021. **58**(4): p. 523-536.

349. Zhang, Y.Y., et al., *Effectiveness of animal-assisted therapy on pain in children: A systematic review and meta-analysis.* INTERNATIONAL JOURNAL OF NURSING SCIENCES, 2021. **8**(1): p. 30-37.

350. Zhao, H., J.C. Hu, and L. Zhao, *Adjunctive subgingival application of Chlorhexidine gel in nonsurgical periodontal treatment for chronic periodontitis: a systematic review and meta-analysis.* BMC ORAL HEALTH, 2020. **20**(1).

351. Zhao, Z.K., et al., *Efficacy evaluation of nilotinib treatment in different genomic subtypes of gastrointestinal stromal tumors: A meta-analysis and systematic review.* CURRENT PROBLEMS IN CANCER, 2021. **45**(3).

352. Zhou, G.P., et al., *Early transjugular intrahepatic portosystemic shunt for acute variceal bleeding: a systematic review and meta-analysis.* EUROPEAN RADIOLOGY, 2021. **31**(7): p. 5390-5399.

353. Zhu, D.Q., et al., *Comparison of Outcomes Between Transperitoneal and Retroperitoneal Robotic Partial Nephrectomy: A Meta-Analysis Based on Comparative Studies.* FRONTIERS IN ONCOLOGY, 2021. **10**.

354. Ziogas, I.A., et al., *The Role of Immunotherapy in Hepatocellular Carcinoma: A Systematic Review and Pooled Analysis of 2,402 Patients.* ONCOLOGIST, 2021. **26**(6): p. E1036-E1049.

355. Ziogas, I.A., et al., *Liver Transplantation for Langerhans Cell Histiocytosis: A US Population-Based Analysis and Systematic Review of the Literature.* LIVER TRANSPLANTATION, 2021. **27**(8): p. 1181-1190.

356. Zuniga, R.A.A., et al., *Clinical effectiveness of drugs in hospitalized patients with COVID-19: a systematic review and meta-analysis.* THERAPEUTIC ADVANCES IN RESPIRATORY DISEASE, 2021. **15**.

357. Zuniga, R.A.A., et al., *Clinical effectiveness of convalescent plasma in hospitalized patients with COVID-19: a systematic review and meta-analysis.* THERAPEUTIC ADVANCES IN RESPIRATORY DISEASE, 2021. **15**.
