# Supplementary material for: The score after 10 years of registration of systematic review protocols
Source: Syst Rev. 2022 Sep 5;11:191. doi: 10.1186/s13643-022-02053-9 (PMC9444273; doi:10.1186/s13643-022-02053-9)
Supplement: Supplementary file 2 — Additional file 2: Supplementary Figure 1. Countries of origen of the corresponding author. Supplementary Table 2. Frequency of included publications per journal title. Supplementary Table 3. outcomes of logistic regression. Supplementary Table 4. Frequency of SR protocols published in journals/ databases other than protocol registries. [file 13643_2022_2053_MOESM2_ESM.docx]

Supplementary file 2

Supplementary Figure 1 – Countries of origin of the corresponding author.


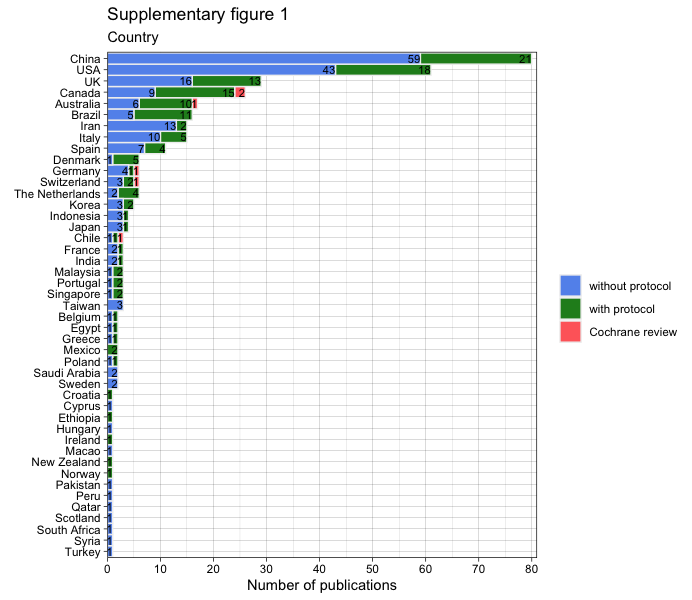


Supplementary table 2 – Frequency of included publications per journal title.

| **Number of publications** | **Journal name** |
| --- | --- |
| 6 | COCHRANE DATABASE OF SYSTEMATIC REVIEWS* |
| 6 | FRONTIERS IN ONCOLOGY |
| 4 | BMJ OPEN* |
| 4 | CRITICAL REVIEWS IN ONCOLOGY HEMATOLOGY |
| 3 | ARTHROSCOPY-THE JOURNAL OF ARTHROSCOPIC AND RELATED SURGERY |
| 3 | CLINICAL ORAL INVESTIGATIONS |
| 3 | COMPLEMENTARY THERAPIES IN MEDICINE |
| 3 | FRONTIERS IN PHARMACOLOGY |
| 3 | JOURNAL OF CLINICAL NEUROSCIENCE |
| 3 | JOURNAL OF THE AMERICAN PHARMACISTS ASSOCIATION |
| 3 | MEDICINE |
| 3 | PHYTOTHERAPY RESEARCH |
| 3 | PLOS ONE* |
| 3 | THERAPEUTIC ADVANCES IN RESPIRATORY DISEASE |
| 3 | WORLD NEUROSURGERY |
| 2 | ADVANCES IN NUTRITION |
| 2 | AMERICAN JOURNAL OF RHINOLOGY & ALLERGY |
| 2 | ARCHIVES OF PHYSICAL MEDICINE AND REHABILITATION |
| 2 | BMC ORAL HEALTH* |
| 2 | CARDIOVASCULAR REVASCULARIZATION MEDICINE |
| 2 | CHEST |
| 2 | CLINICAL AND APPLIED THROMBOSIS-HEMOSTASIS |
| 2 | CLINICAL OTOLARYNGOLOGY |
| 2 | CLINICAL REHABILITATION |
| 2 | CLINICAL RHEUMATOLOGY |
| 2 | CRITICAL REVIEWS IN FOOD SCIENCE AND NUTRITION |
| 2 | DERMATOLOGIC SURGERY |
| 2 | DERMATOLOGIC THERAPY |
| 2 | EFORT OPEN REVIEWS |
| 2 | EUROPEAN JOURNAL OF OBSTETRICS & GYNECOLOGY AND REPRODUCTIVE BIOLOGY |
| 2 | EUROPEAN JOURNAL OF ORTHODONTICS |
| 2 | EVIDENCE-BASED COMPLEMENTARY AND ALTERNATIVE MEDICINE |
| 2 | FRONTIERS IN HUMAN NEUROSCIENCE |
| 2 | FRONTIERS IN PSYCHIATRY |
| 2 | FRONTIERS IN PSYCHOLOGY |
| 2 | INTEGRATIVE CANCER THERAPIES |
| 2 | INTERNATIONAL JOURNAL OF PEDIATRIC OTORHINOLARYNGOLOGY |
| 2 | INTERNATIONAL JOURNAL OF SURGERY |
| 2 | JOURNAL OF ADVANCED NURSING |
| 2 | JOURNAL OF AFFECTIVE DISORDERS |
| 2 | JOURNAL OF CANCER |
| 2 | JOURNAL OF CLINICAL PHARMACY AND THERAPEUTICS |
| 2 | JOURNAL OF PROSTHETIC DENTISTRY |
| 2 | LIFE SCIENCES |
| 2 | MEDICINA-LITHUANIA |
| 2 | NEUROSCIENCE AND BIOBEHAVIORAL REVIEWS |
| 2 | PANCREATOLOGY |
| 2 | PHARMACOLOGICAL RESEARCH |
| 2 | PSYCHOPHARMACOLOGY |
| 2 | REPRODUCTIVE BIOMEDICINE ONLINE |
| 2 | SCIENTIFIC REPORTS |
| 2 | SEMINARS IN ARTHRITIS AND RHEUMATISM |
| 2 | TRANSLATIONAL ANDROLOGY AND UROLOGY |
| 2 | VACCINE |
| 1 | ABCD-ARQUIVOS BRASILEIROS DE CIRURGIA DIGESTIVA-BRAZILIAN ARCHIVES OF DIGESTIVE SURGERY |
| 1 | ACTA ANAESTHESIOLOGICA SCANDINAVICA |
| 1 | ACTA NEUROLOGICA SCANDINAVICA |
| 1 | ACTA OBSTETRICIA ET GYNECOLOGICA SCANDINAVICA |
| 1 | ACTA ONCOLOGICA |
| 1 | ADDICTION |
| 1 | ADDICTIVE BEHAVIORS |
| 1 | ADVANCES IN THERAPY |
| 1 | AMERICAN JOURNAL OF CARDIOLOGY |
| 1 | AMERICAN JOURNAL OF EMERGENCY MEDICINE |
| 1 | AMERICAN JOURNAL OF HEMATOLOGY |
| 1 | AMERICAN JOURNAL OF OBSTETRICS AND GYNECOLOGY |
| 1 | AMERICAN JOURNAL OF OTOLARYNGOLOGY |
| 1 | AMERICAN JOURNAL OF SPORTS MEDICINE |
| 1 | AMERICAN SURGEON |
| 1 | ANNALS OF GASTROENTEROLOGY |
| 1 | ANNALS OF MEDICINE AND SURGERY |
| 1 | ANNALS OF PLASTIC SURGERY |
| 1 | ANNALS OF SURGERY |
| 1 | ANNALS OF THE NEW YORK ACADEMY OF SCIENCES |
| 1 | ANNALS OF THORACIC SURGERY |
| 1 | ANTIBIOTICS-BASEL |
| 1 | ANZ JOURNAL OF SURGERY |
| 1 | ARCHIVES OF GERONTOLOGY AND GERIATRICS |
| 1 | ARCHIVES OF GYNECOLOGY AND OBSTETRICS |
| 1 | ASIAN JOURNAL OF SURGERY |
| 1 | BALKAN MEDICAL JOURNAL |
| 1 | BIOSCIENCE REPORTS |
| 1 | BJPSYCH OPEN |
| 1 | BJS OPEN |
| 1 | BMC COMPLEMENTARY MEDICINE AND THERAPIES* |
| 1 | BMC HEALTH SERVICES RESEARCH* |
| 1 | BMC INFECTIOUS DISEASES* |
| 1 | BMC MEDICAL EDUCATION* |
| 1 | BMC MEDICAL INFORMATICS AND DECISION MAKING* |
| 1 | BMC SURGERY* |
| 1 | BRACHYTHERAPY |
| 1 | BRAIN INJURY |
| 1 | BRITISH JOURNAL OF DERMATOLOGY |
| 1 | BRITISH JOURNAL OF GENERAL PRACTICE |
| 1 | BRITISH JOURNAL OF SURGERY |
| 1 | CANADIAN JOURNAL OF ANESTHESIA-JOURNAL CANADIEN D ANESTHESIE |
| 1 | CANCER CONTROL |
| 1 | CANCER RADIOTHERAPIE |
| 1 | CARDIORENAL MEDICINE |
| 1 | CARDIOVASCULAR AND INTERVENTIONAL RADIOLOGY |
| 1 | CATHETERIZATION AND CARDIOVASCULAR INTERVENTIONS |
| 1 | CEREBROVASCULAR DISEASES |
| 1 | CHILDHOOD OBESITY |
| 1 | CHINESE MEDICINE* |
| 1 | CLINICAL AND EXPERIMENTAL DERMATOLOGY |
| 1 | CLINICAL AND EXPERIMENTAL PHARMACOLOGY AND PHYSIOLOGY |
| 1 | CLINICAL NUTRITION |
| 1 | CLINICAL NUTRITION ESPEN |
| 1 | CLINICAL NUTRITION RESEARCH |
| 1 | CLINICAL PSYCHOLOGY REVIEW |
| 1 | CNS SPECTRUMS |
| 1 | COMMUNITY MENTAL HEALTH JOURNAL |
| 1 | COMPLEMENTARY THERAPIES IN CLINICAL PRACTICE |
| 1 | CRANIO-THE JOURNAL OF CRANIOMANDIBULAR & SLEEP PRACTICE |
| 1 | CRITICAL CARE* |
| 1 | CRITICAL CARE NURSING QUARTERLY |
| 1 | CURRENT PROBLEMS IN CANCER |
| 1 | CURRENT PROBLEMS IN CARDIOLOGY |
| 1 | DENTAL AND MEDICAL PROBLEMS |
| 1 | DEVELOPMENTAL MEDICINE AND CHILD NEUROLOGY |
| 1 | DIABETES & METABOLIC SYNDROME-CLINICAL RESEARCH & REVIEWS |
| 1 | DIABETES OBESITY & METABOLISM |
| 1 | DIABETES THERAPY |
| 1 | DIABETES-METABOLISM RESEARCH AND REVIEWS |
| 1 | DRUG DEVELOPMENT AND INDUSTRIAL PHARMACY |
| 1 | EJSO |
| 1 | ENDOCRINE |
| 1 | ENDOCRINOLOGY DIABETES & METABOLISM |
| 1 | ESC HEART FAILURE |
| 1 | EUROPACE |
| 1 | EUROPEAN JOURNAL OF CANCER |
| 1 | EUROPEAN JOURNAL OF CLINICAL PHARMACOLOGY |
| 1 | EUROPEAN JOURNAL OF CONTRACEPTION AND REPRODUCTIVE HEALTH CARE |
| 1 | EUROPEAN JOURNAL OF HAEMATOLOGY |
| 1 | EUROPEAN JOURNAL OF INTERNAL MEDICINE |
| 1 | EUROPEAN JOURNAL OF ORTHOPAEDIC SURGERY AND TRAUMATOLOGY |
| 1 | EUROPEAN RADIOLOGY |
| 1 | EUROPEAN RESPIRATORY JOURNAL |
| 1 | EUROPEAN RESPIRATORY REVIEW |
| 1 | EVALUATION & THE HEALTH PROFESSIONS |
| 1 | EXPERIMENTAL AND THERAPEUTIC MEDICINE |
| 1 | EXPERT OPINION ON PHARMACOTHERAPY |
| 1 | EXPERT REVIEW OF ANTI-INFECTIVE THERAPY |
| 1 | EXPERT REVIEW OF CLINICAL PHARMACOLOGY |
| 1 | EXPERT REVIEW OF NEUROTHERAPEUTICS |
| 1 | EXPLORE-THE JOURNAL OF SCIENCE AND HEALING |
| 1 | EYE |
| 1 | FERTILITY AND STERILITY |
| 1 | FOOD RESEARCH INTERNATIONAL |
| 1 | FOOT & ANKLE INTERNATIONAL |
| 1 | FRONTIERS IN CARDIOVASCULAR MEDICINE |
| 1 | FRONTIERS IN MEDICINE |
| 1 | FRONTIERS IN PHYSIOLOGY |
| 1 | FRONTIERS IN PUBLIC HEALTH |
| 1 | FRONTIERS IN SURGERY |
| 1 | GENERAL HOSPITAL PSYCHIATRY |
| 1 | GYNECOLOGIC ONCOLOGY |
| 1 | HAEMATOLOGICA |
| 1 | HEALTH TECHNOLOGY ASSESSMENT |
| 1 | HORMONES-INTERNATIONAL JOURNAL OF ENDOCRINOLOGY AND METABOLISM |
| 1 | HRB OPEN RESEARCH |
| 1 | HUMAN FERTILITY |
| 1 | INTERNAL AND EMERGENCY MEDICINE |
| 1 | INTERNATIONAL ARCHIVES OF OTORHINOLARYNGOLOGY |
| 1 | INTERNATIONAL FORUM OF ALLERGY & RHINOLOGY |
| 1 | INTERNATIONAL JOURNAL OF CARDIOLOGY |
| 1 | INTERNATIONAL JOURNAL OF CLINICAL AND HEALTH PSYCHOLOGY |
| 1 | INTERNATIONAL JOURNAL OF COLORECTAL DISEASE |
| 1 | INTERNATIONAL JOURNAL OF ENVIRONMENTAL RESEARCH AND PUBLIC HEALTH |
| 1 | INTERNATIONAL JOURNAL OF INFECTIOUS DISEASES |
| 1 | INTERNATIONAL JOURNAL OF NURSING SCIENCES |
| 1 | INTERNATIONAL JOURNAL OF NURSING STUDIES |
| 1 | INTERNATIONAL JOURNAL OF ORAL AND MAXILLOFACIAL SURGERY |
| 1 | INTERNATIONAL JOURNAL OF PAEDIATRIC DENTISTRY |
| 1 | INTERNATIONAL ORTHOPAEDICS |
| 1 | JAMA INTERNAL MEDICINE |
| 1 | JAPAN JOURNAL OF NURSING SCIENCE |
| 1 | JBI EVIDENCE SYNTHESIS* |
| 1 | JOURNAL OF ADOLESCENT AND YOUNG ADULT ONCOLOGY |
| 1 | JOURNAL OF ANTIMICROBIAL CHEMOTHERAPY |
| 1 | JOURNAL OF APPLIED ORAL SCIENCE |
| 1 | JOURNAL OF ARRHYTHMIA |
| 1 | JOURNAL OF ASTHMA |
| 1 | JOURNAL OF ATHLETIC TRAINING |
| 1 | JOURNAL OF BODYWORK AND MOVEMENT THERAPIES |
| 1 | JOURNAL OF CANNABIS RESEARCH |
| 1 | JOURNAL OF CARDIOVASCULAR ELECTROPHYSIOLOGY |
| 1 | JOURNAL OF CARDIOVASCULAR MEDICINE |
| 1 | JOURNAL OF CARDIOVASCULAR NURSING |
| 1 | JOURNAL OF CARDIOVASCULAR PHARMACOLOGY |
| 1 | JOURNAL OF CLINICAL MEDICINE |
| 1 | JOURNAL OF COMMUNITY HOSPITAL INTERNAL MEDICINE PERSPECTIVES |
| 1 | JOURNAL OF CUTANEOUS MEDICINE AND SURGERY |
| 1 | JOURNAL OF DENTAL SCIENCES |
| 1 | JOURNAL OF DERMATOLOGICAL TREATMENT |
| 1 | JOURNAL OF DEVELOPMENTAL ORIGINS OF HEALTH AND DISEASE |
| 1 | JOURNAL OF DIETARY SUPPLEMENTS |
| 1 | JOURNAL OF ETHNOPHARMACOLOGY |
| 1 | JOURNAL OF EVIDENCE-BASED DENTAL PRACTICE |
| 1 | JOURNAL OF GASTROINTESTINAL SURGERY |
| 1 | JOURNAL OF GENERAL INTERNAL MEDICINE |
| 1 | JOURNAL OF GERIATRIC PHYSICAL THERAPY |
| 1 | JOURNAL OF HOSPITAL INFECTION |
| 1 | JOURNAL OF INTERNATIONAL SOCIETY OF PREVENTIVE AND COMMUNITY DENTISTRY |
| 1 | JOURNAL OF INVESTIGATIVE MEDICINE HIGH IMPACT CASE REPORTS |
| 1 | JOURNAL OF INVESTIGATIVE SURGERY |
| 1 | JOURNAL OF LAPAROENDOSCOPIC & ADVANCED SURGICAL TECHNIQUES |
| 1 | JOURNAL OF MATERNAL-FETAL & NEONATAL MEDICINE |
| 1 | JOURNAL OF NEPHROLOGY |
| 1 | JOURNAL OF NEUROENGINEERING AND REHABILITATION* |
| 1 | JOURNAL OF NEUROLOGY NEUROSURGERY AND PSYCHIATRY* |
| 1 | JOURNAL OF NURSING SCHOLARSHIP |
| 1 | JOURNAL OF OBSTETRICS AND GYNAECOLOGY CANADA |
| 1 | JOURNAL OF OBSTETRICS AND GYNAECOLOGY RESEARCH |
| 1 | JOURNAL OF ORAL REHABILITATION |
| 1 | JOURNAL OF ORTHOPAEDIC SCIENCE |
| 1 | JOURNAL OF ORTHOPAEDIC SURGERY AND RESEARCH* |
| 1 | JOURNAL OF ORTHOPAEDICS |
| 1 | JOURNAL OF PAIN |
| 1 | JOURNAL OF PALLIATIVE MEDICINE |
| 1 | JOURNAL OF PARENTERAL AND ENTERAL NUTRITION |
| 1 | JOURNAL OF PEDIATRIC SURGERY |
| 1 | JOURNAL OF PERSONALIZED MEDICINE |
| 1 | JOURNAL OF PLASTIC RECONSTRUCTIVE AND AESTHETIC SURGERY |
| 1 | JOURNAL OF PSYCHIATRIC RESEARCH |
| 1 | JOURNAL OF SHOULDER AND ELBOW SURGERY |
| 1 | JOURNAL OF SUBSTANCE ABUSE TREATMENT |
| 1 | JOURNAL OF THE INTERNATIONAL ACADEMY OF PERIODONTOLOGY |
| 1 | JOURNAL OF THE INTERNATIONAL AIDS SOCIETY |
| 1 | JOURNAL OF THROMBOSIS AND THROMBOLYSIS |
| 1 | JOURNAL OF TRANSLATIONAL MEDICINE* |
| 1 | JOURNAL OF VASCULAR SURGERY |
| 1 | JOURNAL OF VASCULAR SURGERY-VENOUS AND LYMPHATIC DISORDERS |
| 1 | JOURNAL OF VOICE |
| 1 | KOREAN JOURNAL OF PAIN |
| 1 | LANCET HIV |
| 1 | LARYNGOSCOPE |
| 1 | LIVER TRANSPLANTATION |
| 1 | MATERNAL AND CHILD NUTRITION |
| 1 | MATURITAS |
| 1 | MEDICAL DECISION MAKING |
| 1 | METABOLIC BRAIN DISEASE |
| 1 | MINERVA ANESTESIOLOGICA |
| 1 | MODERN RHEUMATOLOGY |
| 1 | MUSCULOSKELETAL SCIENCE AND PRACTICE |
| 1 | NEURO-ONCOLOGY ADVANCES |
| 1 | NEUROLOGICAL SCIENCES |
| 1 | NEURORADIOLOGY |
| 1 | NUTRITION METABOLISM AND CARDIOVASCULAR DISEASES |
| 1 | NUTRITIONAL NEUROSCIENCE |
| 1 | ONCOLOGIST |
| 1 | OTOLARYNGOLOGY-HEAD AND NECK SURGERY |
| 1 | PACE-PACING AND CLINICAL ELECTROPHYSIOLOGY |
| 1 | PEDIATRIC RESEARCH |
| 1 | PEDIATRIC SURGERY INTERNATIONAL |
| 1 | PEDIATRICS |
| 1 | PERITONEAL DIALYSIS INTERNATIONAL |
| 1 | PERSPECTIVES IN PSYCHIATRIC CARE |
| 1 | PHOTODIAGNOSIS AND PHOTODYNAMIC THERAPY |
| 1 | PROGRESS IN ORTHODONTICS |
| 1 | REGIONAL ANESTHESIA AND PAIN MEDICINE* |
| 1 | RESUSCITATION |
| 1 | RETINA-THE JOURNAL OF RETINAL AND VITREOUS DISEASES |
| 1 | RMD OPEN* |
| 1 | SCANDINAVIAN JOURNAL OF MEDICINE & SCIENCE IN SPORTS |
| 1 | SLEEP MEDICINE REVIEWS |
| 1 | SPORTS HEALTH-A MULTIDISCIPLINARY APPROACH |
| 1 | SPORTS MEDICINE |
| 1 | STEM CELL RESEARCH & THERAPY* |
| 1 | SUPPORTIVE CARE IN CANCER |
| 1 | SURGERY FOR OBESITY AND RELATED DISEASES |
| 1 | SURGICAL ENDOSCOPY AND OTHER INTERVENTIONAL TECHNIQUES |
| 1 | SURGICAL ONCOLOGY |
| 1 | SYSTEMATIC REVIEWS* |
| 1 | TECHNIQUES IN COLOPROCTOLOGY |
| 1 | THORAX* |
| 1 | THROMBOSIS AND HAEMOSTASIS |
| 1 | TRANSPLANTATION PROCEEDINGS |
| 1 | VASA-EUROPEAN JOURNAL OF VASCULAR MEDICINE |
| 1 | VASCULAR |

*journal or journal publisher has endorsed PROSPERO as reported on PROSPERO website (https://www.crd.york.ac.uk/prospero/#aboutpage)

Supplementary table 3 – outcomes of logistic regression

|  | **Estimate** | **SE** | **OR** | **95%CI lower bound** | **95%CI upper bound** |
| --- | --- | --- | --- | --- | --- |
| (Intercept) | -0.82041 | 0.80896 | NA | NA | NA |
| **Number of authors** | -0.04025 | 0.05498 | 0.96 | 0.86 | 1.06 |
| **Number of affiliations** | 0.02806 | 0.05366 | 1.02 | 0.93 | 1.14 |
| **Journal impact factor** | 0.13099 | 0.04808 | **1.12** | 1.04 | 1.25 |
| **Funding** |  | | | | |
| *No funding* | Reference category | | | | |
| *For profit* | -0.44794 | 0.89015 | 0.64 | 0.11 | 3.65 |
| *Nonprofit* | 0.11685 | 0.29772 | 1.12 | 0.63 | 2.01 |
| *Not clear* | -0.29424 | 0.81059 | 0.75 | 0.15 | 365 |
| *Not reported* | -0.29177 | 0.31246 | 0.75 | 0.40 | 1.38 |
| **COI statement** |  | | | | |
| *No* | Reference category | | | | |
| *Yes* | -0.89409 | 0.60824 | 0.41 | 0.12 | 1.34 |
| **PRISMA** |  | | | | |
| *No* | Reference category | | | | |
| *Yes* | 0.99724 | 0.41325 | **2.71** | 1.21 | 6.09 |
| *only in flow-chart* | 0.52267 | 0.56556 | 1.69 | 0.56 | 5.11 |
| **Year** |  | | | | |
| *2020* | Reference category | | | | |
| *2021* | 0.42262 | 0.24195 | 1.53 | 0.95 | 2.45 |
| **Continent** |  | | | | |
| *Europe* | Reference category | | | | |
| *Africa* | -0.06087 | 1.05510 | 0.94 | 0.12 | 7.44 |
| *Asia* | -0.84271 | 0.31371 | **0.43** | 0.23 | 0.80 |
| *Australia* | 0.72588 | 0.58787 | 2.07 | 0.65 | 6.54 |
| *North America* | 0.02565 | 0.31498 | 1.02 | 0.55 | 1.90 |
| *South America* | 0.95669 | 0.55048 | 2.60 | 0.88 | 7.66 |

Abbreviations: SE=Standard Error, OR=Odds Ratio, NA=Not applicable, JIF=Journal Impact Factor, COI=conflicts of interest, PRISMA= Preferred Reporting Items for Systematic Reviews and Meta-Analysis

McFadden R2=0.096, p<0.001, Variance Inflation Factor values ranged from 1.05 to 2.26

Supplementary table 4 – Frequency of SR protocols published in journals/ databases other than protocol registries.

| **Number of publications** | **Journal name** |
| --- | --- |
| 7 | COCHRANE DATABASE OF SYSTEMATIC REVIEWS |
| 2 | BMC SYSTEMATIC REVIEWS |
| 1 | BMJ OPEN |
| 1 | JBI DATABASE OF SYSTEMATIC REVIEWS AND IMPLEMENTATION REPORTS |
| **Other type of protocol publication** | |
| 1 | NICE WEBSITE |
